# EGFR- and HER3-targeted bispecific antibody-drug conjugate demonstrates antitumor activity in metastatic castration-resistant prostate cancer

**DOI:** 10.1172/JCI201090

**Published:** 2026-04-07

**Authors:** Bangwei Fang, Xiaomeng Li, Ying Lu, Weiwei Ma, Hualei Gan, Tingwei Zhang, Qi Liu, Beihe Wang, Zixian Wang, Yi Zhu, Hai Zhu, Sa Xiao, Xiaojie Bian, Gonghong Wei, Dingwei Ye, Yao Zhu

**Affiliations:** 1 Department of Urology, Fudan University Shanghai Cancer Center, Shanghai, China.; 2 Department of Oncology, Shanghai Medical College, Fudan University, Shanghai, China.; 3 Shanghai Genitourinary Cancer Institute, Shanghai, China.; 4 Key Laboratory of Metabolism and Molecular Medicine of the Ministry of Education, Department of Biochemistry and Molecular Biology of School of Basic Medical Sciences, Shanghai Medical College of Fudan University, Shanghai, China.; 5 Department of Pathology, Fudan University Shanghai Cancer Center, Shanghai, China.; 6 Sichuan Biokin Pharmaceutical Co., Ltd. Chengdu, China.; 7 Baili-Bio (Chengdu) Pharmaceutical Co., Ltd. Chengdu, China

**Keywords:** Clinical Research, Oncology, Prostate cancer

## Abstract

Metastatic castration-resistant prostate cancer (mCRPC) remains lethal with limited treatment options. Antibody–drug conjugates (ADCs) have emerged as a transformative class across multiple solid tumors, yet their clinical application in prostate cancer has been limited. Izalontamab brengitecan (Iza-bren; BL-B01D1) is a bispecific ADC-targeting EGFR and HER3 that has demonstrated activity in other malignancies. Here, we evaluated its therapeutic potential in the treatment of prostate cancer. Multi-omics analyses revealed frequent EGFR and HER3 expression in CRPC adenocarcinoma but not in neuroendocrine subtypes. BL-B01D1 exerted potent, target-dependent cytotoxicity in prostate cancer cell lines, xenografts, and patient-derived organoids (PDOs). We highlight a representative patient with mCRPC with high EGFR/HER3 expression whose disease rapidly and durably mounted a clinical and radiologic response to BL-B01D1, concordant with matched PDO sensitivity. Mechanistic studies identified ABCG2 upregulation as a driver of acquired resistance, with genetic or pharmacologic inhibition restoring BL-B01D1 sensitivity. Importantly, tumor tissue obtained at progression after BL-B01D1 treatment confirmed ABCG2 upregulation, validating a clinically relevant resistance mechanism. These findings support BL-B01D1 as a promising therapeutic strategy in mCRPC and indicate ABCG2 may be a rational target for overcoming resistance.

## Abstract 2

A targeted therapy against two specific proteins delivers a cytotoxic payload, demonstrating effectiveness in advanced prostate cancer.

## Introduction

Prostate cancer is the second most common malignancy in men worldwide and a leading cause of cancer-related death (1). Although androgen deprivation therapy (ADT) is effective in the majority of patients with advanced prostate cancer, the disease in almost all will eventually progress to metastatic castration-resistant prostate cancer (mCRPC) (2, 3). Following resistance to next-generation androgen receptor (AR) signaling inhibitors and chemotherapy, treatment options for these patients remain limited, with a median overall survival of 14–15 months (4, 5).

Previous studies have highlighted the critical role of the epidermal growth factor (EGF) signaling pathway in the progression of prostate cancer. Elevated EGFR expression promotes metastasis of prostate cancer (6, 7), whereas activation of HER2/HER3 downstream signaling maintains AR protein stability and contributes to the development of resistance to AR inhibitors (8–11). However, clinical trials of EGF pathway–targeted therapies have shown limited antitumor effects in patients with advanced prostate cancer (12–14), leading to the discontinuation of targeting the EGF pathway as a treatment strategy for prostate cancer.

The rise of antibody-drug conjugates (ADCs) is revolutionizing the cancer treatment landscape, offering an opportunity to reassess the potential of previously explored tumor targets (15, 16). ADCs have shown remarkable success in several malignancies, particularly in breast cancer, non–small-cell lung cancer and urothelial cancer, exhibiting substantial clinical efficacy and improved therapeutic outcomes (17–19). However, despite the encouraging progress in other cancer types, the application of ADCs in prostate cancer remains largely investigational and has not yet received regulatory approval (20). Izalontamab brengitecan (Iza-bren; BL-B01D1) is a first-in-class EGFR-HER3 bispecific ADC consisting of a bispecific antibody, a cleavable tetrapeptide linker, and the cytotoxic agent Ed-04 (a camptothecin-derivative topoisomerase I inhibitor) (21). The drug-to-antibody ratio is 8. Compared with monospecific ADCs, bispecific ADCs offer distinct advantages by simultaneously targeting 2 different antigens, which may enhance internalization, overcome tumor heterogeneity, and reduce the probability of resistance development (22, 23). BL-B01D1 has shown potent antitumor activity in various solid tumors, including lung cancer, nasopharyngeal cancer, and esophageal squamous cell carcinoma (24, 25), but its antitumor effects in prostate cancer remain unexplored.

Given the considerable dependence of ADC efficacy on the expression abundance of the targeted antigens in tumors, it is essential to assess the expression patterns of these targets in specimens from clinical patients. In this study, we comprehensively characterized the expression profiles of EGFR and HER3 in prostate cancer using publicly available omics data and IHC analysis of patient samples. Additionally, we evaluated the antitumor activity of BL-B01D1 in several preclinical prostate cancer models, including patient-derived organoids (PDOs), and described a representative clinical case of a patient with mCRPC treated with BL-B01D1 on an ongoing clinical trial (ClinicalTrials.gov NCT05785039). Finally, we conducted preliminary investigations on the mechanisms of resistance to BL-B01D1 in prostate cancer and identified ABCG2 as a potential targetable resistance-related protein.

## Results

### Genomic alterations and mRNA expression of EGFR and ERBB3 in patients with prostate cancer.

**Figure 1:**
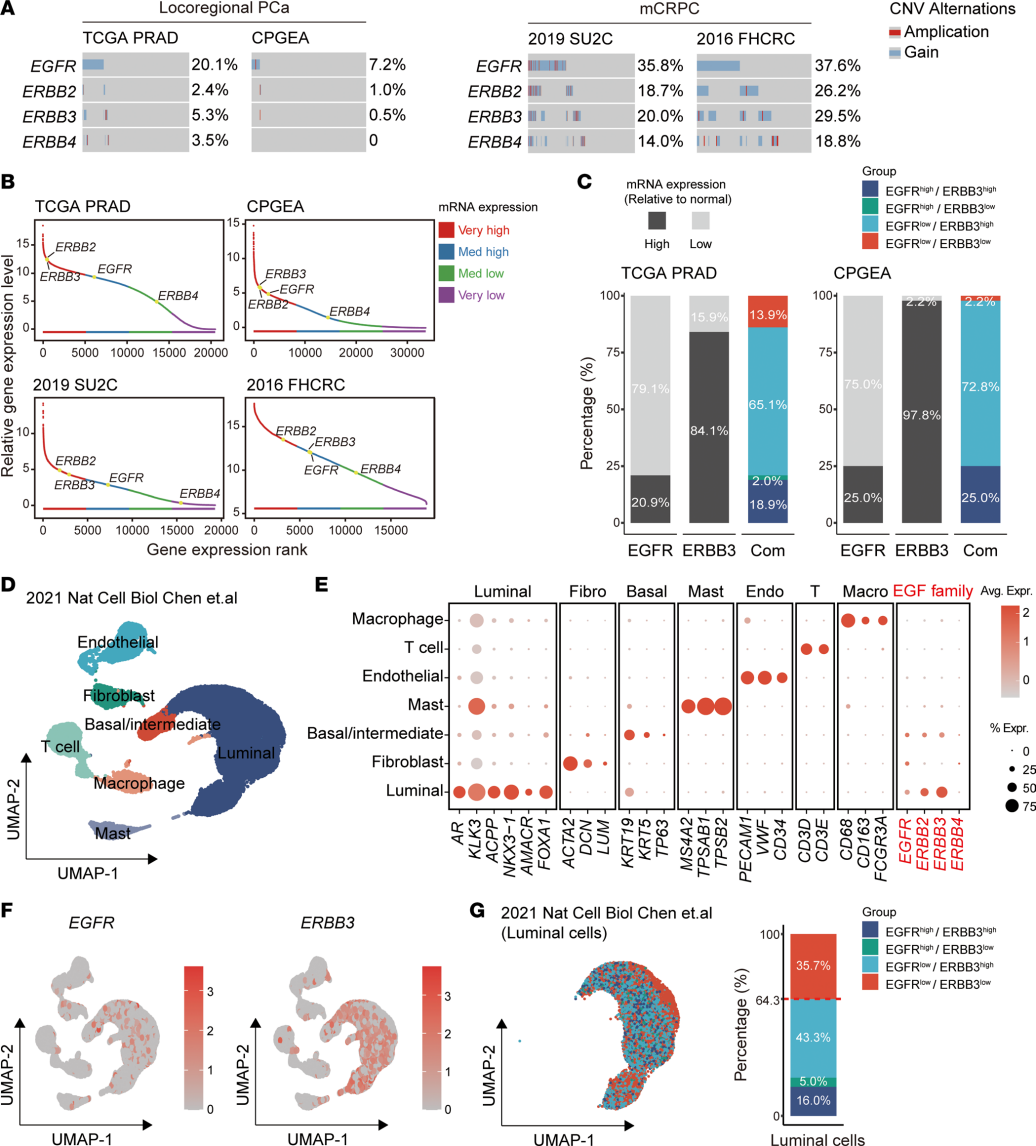
Genomic alterations and mRNA expression pattern of *EGFR* and *ERBB3* in prostate cancer. (**A**) Frequency of genomic gain and amplification of ErbB family genes (*EGFR*, *ERBB2*, *ERBB3*, *ERBB4*) across 4 prostate cancer cohorts, including localized prostate cancer (TCGA and CPGEA) and mCRPC (SU2C and FHCRC). CNV, copy number variation. (**B**) Relative mRNA expression ranking of EGFR family members in 4 prostate cancer cohorts. (**C**) Proportion of tumor samples with high or low *EGFR* and *ERBB3* expression in TCGA and CPGEA datasets. Com, combined. (**D**) Uniform manifold approximation and projection (UMAP) visualization of cell-type clusters from a published scRNA-Seq dataset of 13 patients with prostate cancer. Data were obtained from the GEO dataset GSE141445 (ref. 34) and re-analyzed in this study. (**E**) Dot plot showing the RNA expression levels of representative marker genes and ErbB family genes in annotated cell clusters. Avg. Expr., average expression. % Expr., percentage of expression. (**F**) Feature plots displaying the spatial distribution and relative expression levels of *EGFR* and *ERBB3* across all the cell clusters. (**G**) Proportional distribution of luminal tumor cells classified by *EGFR* and *ERBB3* expression levels.

To evaluate the potential clinical applicability of targeting the EGF receptor family as a therapeutic strategy in prostate cancer, we first conducted a comprehensive genomic analysis of localized prostate cancer datasets (The Cancer Genome Atlas [TCGA], *n* = 492; Chinese Prostate Cancer Genome and Epigenome Atlas [CPGEA], *n* = 208) and mCRPC cohorts (Stand Up to Cancer–Mark Foundation [SU2C], *n* = 444; Fred Hutchinson Cancer Research Center [FHCRC], *n* = 149) (26–29). Unlike other malignancies such as lung cancer, colorectal cancer, and head and neck squamous cell carcinoma, where mutations in ErbB family genes (*EGFR*, *ERBB2*, *ERBB3*, and *ERBB4*) are commonly observed and have led to the successful development of targeted therapies (30), such mutations are relatively infrequent in prostate cancer (Supplemental Figure 1A; supplemental material available online with this article; https://doi.org/10.1172/JCI201090DS1). Consequently, we focused our analysis on gene copy number alterations (CNAs), particularly gain/amplification events. Our results demonstrated a markedly higher prevalence of genomic gain and amplification of ErbB family genes in CRPC samples compared with localized disease (Figure 1A). These genomic alterations were accompanied by a corresponding increase in mRNA expression levels (Supplemental Figure 1, B–E), suggesting that copy number–driven overexpression of these receptors may contribute to progression to CRPC.

We next examined the transcriptomic expression of these 4 genes. *EGFR*, *ERBB2*, and *ERBB3* showed relatively high expression levels in both localized prostate cancer and metastatic CRPC tissues, ranking in the top 25% (very high) or top 50% (median high) of overall gene expression levels, whereas *ERBB4* expression was relatively low (Figure 1B). Given that previous studies have reported relative low protein expression levels of *ERBB2* in prostate cancer (31–33), we focused our subsequent analyses on *EGFR* and *ERBB3*. Using 2 transcriptomic datasets (TCGA and CPGEA) that included matched normal prostate tissues, we classified tumor samples as high or low expression based on whether their *EGFR* or *ERBB3* mRNA levels were above or below the median expression in normal tissues. Approximately 20% of prostate cancer tumors showed high *EGFR* expression (TCGA: 20.9%; CPGEA: 25.0%), whereas nearly 90% exhibited high *ERBB3* expression (TCGA: 84.1%; CPGEA: 97.8%). Most tumors showed high *ERBB3* expression alone (TCGA: 65.1%; CPGEA: 72.8%), but a small fraction exhibited high *EGFR* expression alone (TCGA: 2.0%; CPGEA: 0%) or low expression of both genes (TCGA: 13.9%; CPGEA: 2.2%) (Figure 1C).

To further investigate *EGFR* and *ERBB3* expression at single-cell resolution, we analyzed a published single-cell RNA-Seq dataset comprising prostate cancer tissues from 13 patients (Figure 1D) (34). *EGFR* and *ERBB3* expression were primarily observed in luminal and basal epithelial cells, with a small proportion of fibroblasts expressing *EGFR* (Figure 1, E and F). We defined high and low expression using the first quartile of global gene expression as the cutoff. Among luminal cells—the primary malignant epithelial cell type in prostate cancer—21.0% of tumor cells were classified as EGFR-high and 59.3% as ERBB3-high, including 16.0% dual high-expression cells, 5.0% EGFR-high only, 43.3% ERBB3-high only, and 35.7% dual low-expression cells (Figure 1G). In summary, these results suggest that *EGFR* and *ERBB3* are commonly and highly expressed in prostate cancer, supporting their potential as therapeutic targets in a remarkable subset (approximately 75%) of patients.

### Heterogeneous expression of EGFR and HER3 in CRPC.

**Figure 2:**
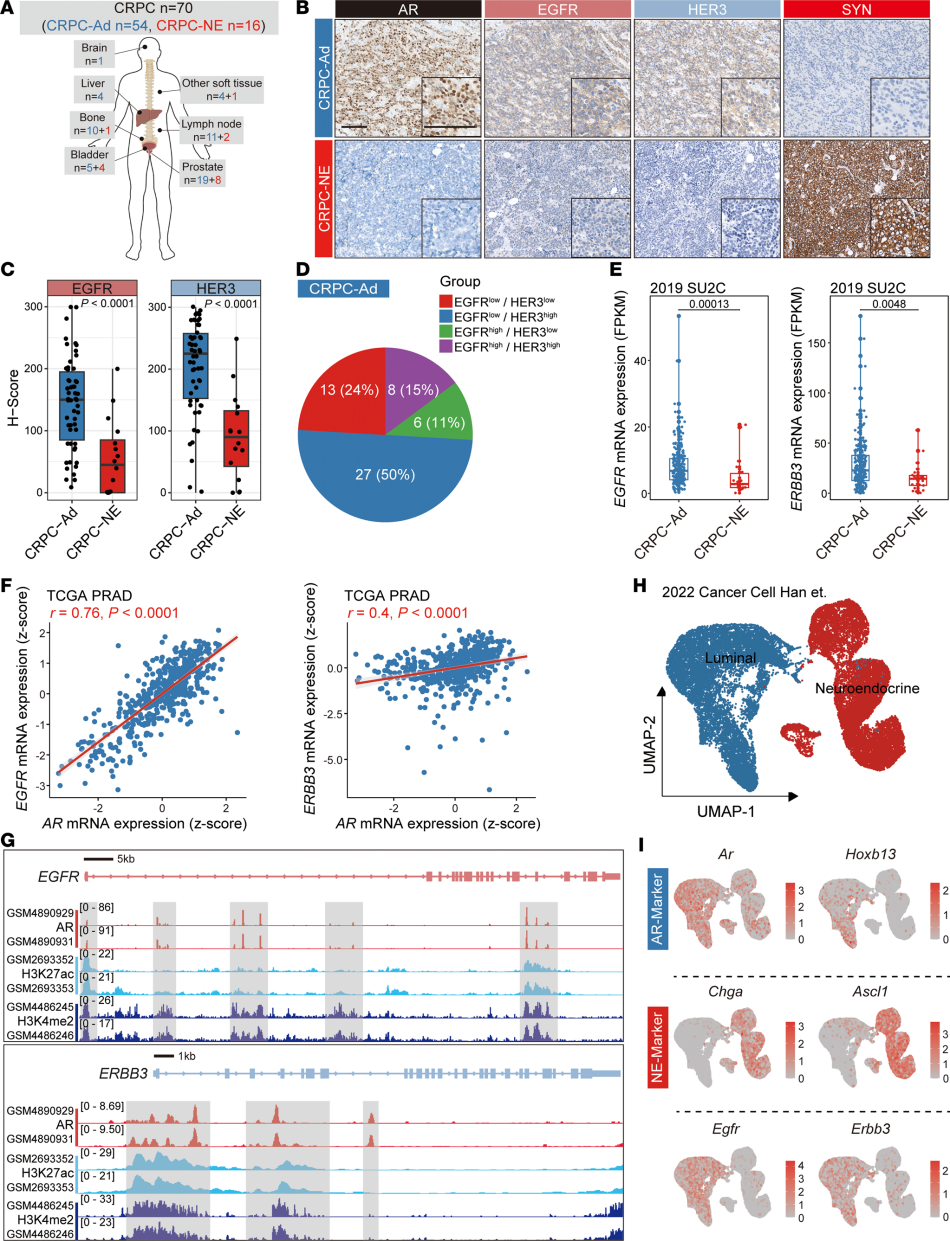
Distinct expression patterns of EGFR and HER3 across CRPC subtypes and their association with AR signaling. (**A**) Schematic overview of specimen sources from 70 patients with CRPC, stratified by CRPC-Ad (*n* = 54) and CRPC-NE (*n* = 16). (**B**) Representative IHC staining of AR, EGFR, HER3, and synaptophysin (SYN) in CRPC-Ad (*n* = 54) and CRPC-NE (*n* = 16) tumors. Scale bar, 100 μm. (**C**) Quantification of EGFR and HER3 protein levels (H-scores) in CRPC-Ad (*n* = 54) and CRPC-NE (*n* = 16) subgroups. (**D**) Distribution of EGFR and HER3 co-expression patterns among CRPC-Ad cases. (**E**) Comparison of *EGFR* and *ERBB3* mRNA expression between CRPC-Ad and CRPC-NE tumors in the SU2C cohort. (**F**) Pearson correlations analysis between *AR* and *EGFR* (left panel) or *ERBB3* (right panel) mRNA expression in the TCGA-PRAD dataset. (**G**) ChIP-Seq tracks showing AR binding peaks and active histone marks (H3K27ac and H3K4me2) at the *EGFR* (upper panel) and *ERBB3* (lower panels) loci. (**H**) Uniform manifold approximation and projection (UMAP) plot of luminal and neuroendocrine cell clusters in an NEPC mouse model scRNA-Seq dataset. (**I**) Feature plots showing mutually exclusive expression of AR markers (*Ar*, *Hoxb13*), NE markers (*Chga*, *Ascl1*), and *Egfr*/*Erbb3* in scRNA-Seq data of the NEPC mouse model. The box plot represents the IQR divided by the median (**C** and **E**). Statistical significance was determined by Mann-Whitney *U* test (**C** and **E**) or Pearson correlation test (**F**).

To investigate EGFR and HER3 protein expression in advanced prostate cancer, we analyzed, by IHC, tumor tissues from 70 patients with CRPC who were treated at Fudan University Shanghai Cancer Center (FUSCC) (Figure 2A), including 10 patients with matched hormone-sensitive prostate cancer (HSPC) samples. This cohort comprised 54 patients with adenocarcinoma phenotype (CRPC-Ad) and 16 with neuroendocrine features (CRPC-NE), the latter of which were characterized by expression of neuroendocrine markers such as SYP and CHGA and loss of AR expression (Figure 2, A and B, and Supplemental Figure 2, A and B) (35). The clinical characteristics of these patients are summarized in Supplemental Table 1. EGFR and HER3 expression levels did not significantly differ across lesions derived from different anatomic sites (Supplemental Figure 2, C–E). Interestingly, we observed differential expression patterns based on histological subtype. In CRPC-Ad, HER3 was highly expressed in 64.8% of tumors (*n* = 35 of 54; median histochemistry score [H-score], 225), and EGFR was highly expressed in 25.9% of cases (*n* = 14 of 54; median H-score, 150) (Figure 2, C and D). In contrast, EGFR and HER3 expression was generally low in CRPC-NE tumors, with high HER3 expression observed in only 6.3% (*n* = 1 of 16) of cases (median H-score = 100) and high EGFR expression detected in 6.3% (*n* = 1 of 16) of cases (median H-score = 50). (Figure 2C and Supplemental Figure 2, F and G). Among patients with CRPC-Ad, 14.8% (*n* = 8 of 54) showed high co-expression of EGFR and HER3, 50.0% (*n* = 27 of 54) had high HER3 expression only, 11.1% (*n* = 6 of 54) had high EGFR expression only, and 24.1% (*n* = 13 of 54) showed low expression of both targets (Figure 2D). In paired samples, EGFR and HER3 expression was mildly increased from HSPC to CRPC stages (median H-score: EGFR: 145 vs. 160, *P* = 0.12; HER3: 170 vs. 205, *P* = 0.54), without statistical significance, likely due to the small sample size (Supplemental Figure 2, H and I).

To validate these findings at the transcriptomic level, we analyzed *EGFR* and *ERBB3* mRNA expression in SU2C cohort samples with known pathology. Expression of both genes was significantly higher in CRPC-Ad compared with CRPC-NE (Figure 2E). Similar trends were observed in the Weill Cornell Medicine cohort, which was enriched for CRPC-NE (36), though statistical significance was not reached, due to limited sample size (Supplemental Figure 3A). Moreover, in both cohorts, samples with high neuroendocrine prostate cancer (NEPC) transcriptomic scores had lower *EGFR* and *ERBB3* mRNA expression (Supplemental Figure 3, B and C), whereas those with high AR signaling had higher expression levels (Supplemental Figure 3, D and E).

Given that loss of AR signaling is a key characteristic of CRPC-NE progression (37), we hypothesized that *EGFR* and *ERBB3* may be transcriptionally regulated by AR. Consistent with this notion, we found strong positive correlations between *AR* and *EGFR* or *ERBB3* mRNA levels in localized prostate cancer (EGFR: *r* = 0.76, *P* < 0.0001; HER3: *r* = 0.4, *P* < 0.0001) (Figure 2F). Analysis of publicly available ChIP-Seq data revealed direct AR binding peaks at both *EGFR* and *ERBB3* loci, including promoter regions, which overlapped with H3K27ac and H3K4me2 active chromatin marks—suggesting that AR may enhance transcription of these genes (Figure 2G). Supporting this, analysis of an independent Gene Expression Omnibus (GEO) dataset (GSE68993) showed that knockdown of AR in CWR22Pc cells, a prostate cancer cell line, resulted in a significant reduction in *EGFR* and *ERBB3* mRNA levels compared with the shRNA control group, further validating AR as a transcriptional regulator of both targets (Supplemental Figure 4, F-H). Finally, using a published scRNA-Seq dataset of a mouse model of neuroendocrine differentiation in prostate cancer (38), we found that *Egfr* and *Erbb3* mRNA expression levels were high in adenocarcinoma cells but were markedly downregulated during NEPC transdifferentiation (Figure 2, H and I, and Supplemental Figure 4, A and B), further supporting their association with AR-mediated transcriptional regulation. Collectively, EGFR and HER3 exhibit distinct expression patterns across pathology subtypes of CRPC, with high expression predominantly observed in CRPC-Ad and markedly reduced levels in CRPC-NE. These differences are consistent at both the protein and transcriptomic levels, and correlate with AR signaling activity.

### Antitumor activity of BL-B01D1 in in vitro and in vivo prostate cancer preclinical models.

**Figure 3:**
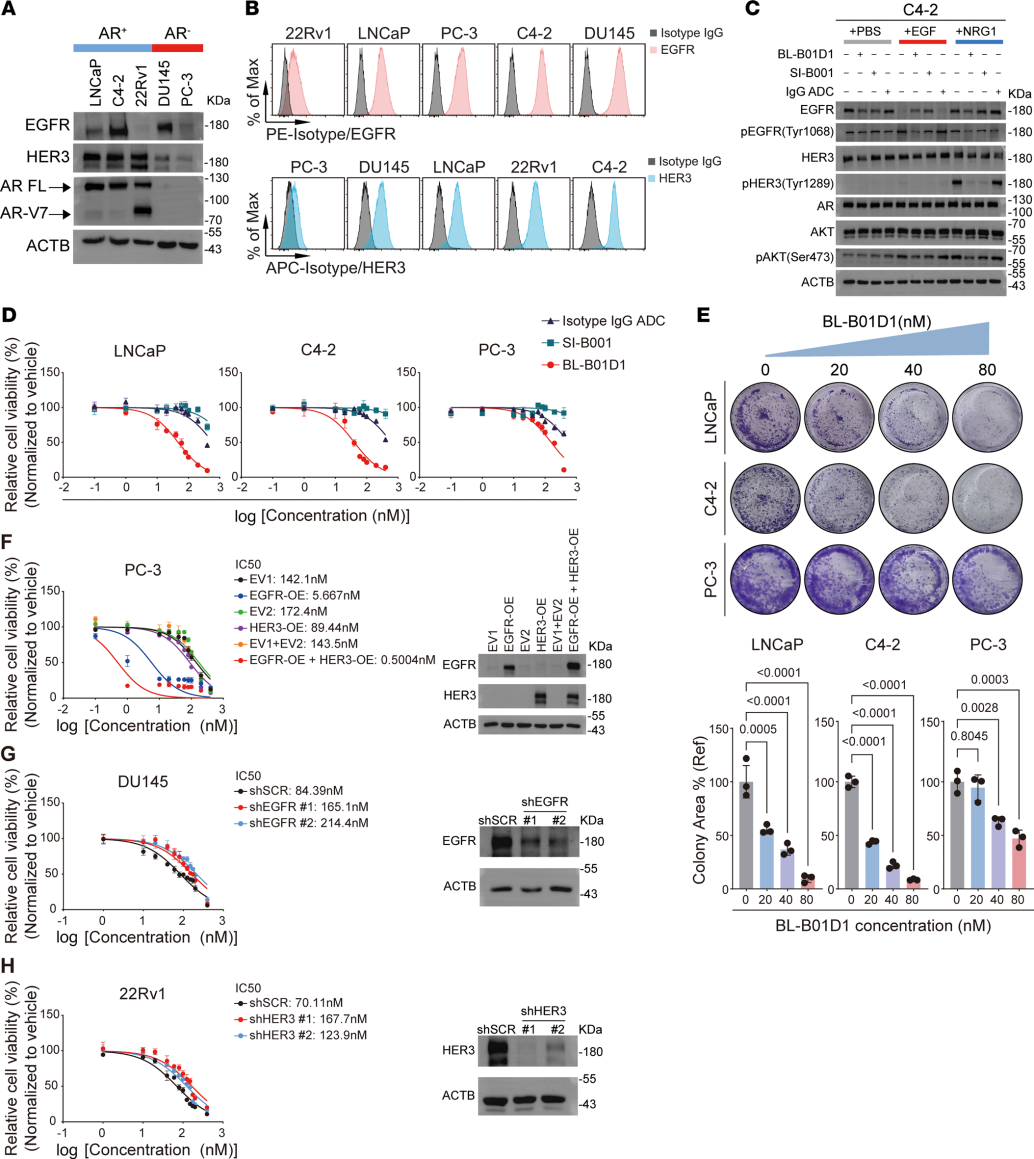
Antitumor activity of BL-B01D1 in human prostate cancer cell line models in vitro. (**A**) Immunoblot of EGFR, HER3, and AR in prostate cancer cell lines. (**B**) Flow cytometry of EGFR/HER3 expression in prostate cancer cell lines. Max, maximum. (**C**) Immunoblot of downstream signaling pathways and AR in C4-2 cells treated with PBS, BL-B01D1, SI-B001, or isotype IgG ADC ± EGF or NRG-1. (**D**) Cell viability assays comparing BL-B01D1, SI-B001, and isotype IgG ADC (*n* = 3). (**E**) Colony formation assays after BL-B01D1 treatment with quantification (*n* = 3). Ref, reference. (**F**) Cell viability and Western blots in PC-3 cells overexpressing EGFR, HER3, or both, after BL-B01D1 treatment (*n* = 3). EV, empty vector. (**G** and **H**) Cell viability assays after BL-B01D1 treatment in DU145 cells with EGFR knockdown (**G**) and 22Rv1 cells with HER3 knockdown (**H**) (*n* = 3), with corresponding Western blots. Data are presented as mean ± SD (**D**–**H**). Statistical significance was determined by 1-way ANOVA with Dunnett’s multiple comparisons (**E**).

We next evaluated the antitumor activity of BL-B01D1, a bispecific ADC that simultaneously targets EGFR and HER3 and is conjugated with a camptothecin-derivative topoisomerase I inhibitor Ed-04 (21), in preclinical models of prostate cancer. First, we assessed the protein expression levels of EGFR and HER3 across a panel of commonly used human prostate cancer cell lines. Western blot analysis revealed that HER3 was highly expressed in all AR-positive cell lines, whereas EGFR showed high expression in C4-2, moderate expression in lymph node carcinoma of the prostate (LNCaP), and minimal expression in 22Rv1 (Figure 3A). In AR-negative cell lines, DU145 exhibited high EGFR expression, whereas both EGFR and HER3 were expressed at low levels in PC-3 cells (Figure 3A). These findings were largely consistent with those obtained from flow cytometry analysis (Figure 3B).

Next, we examined the ability of BL-B01D1 and its unconjugated antibody backbone, SI-B001 (39), to block ligand-induced activation of EGFR and HER3. Solvent and isotype IgG ADC were used as controls. Both BL-B01D1 and SI-B001 effectively inhibited the activation of downstream signaling pathways upon stimulation with exogenous EGF (EGFR ligand) or NRG1 (HER3 ligand) (Figure 3C and Supplemental Figure 5, A and B). Interestingly, in 22Rv1 cells, HER3 blockade led to a reduction in AR protein levels (Supplemental Figure 5B), consistent with previous findings that EGF signaling contributes to AR protein stabilization (8). However, this phenomenon was not observed in C4-2 cells, suggesting that EGF pathway–mediated AR stabilization may be context dependent (Figure 3C).

We then assessed the in vitro cytotoxic activity of BL-B01D1, SI-B001 and the isotype IgG ADC across various prostate cancer cell lines. BL-B01D1 demonstrated the most potent antitumor effect in C4-2 cells, which co-express EGFR and HER3 at high levels (Figure 3, D and E, and Supplemental Figure 5, C–E). It also exerted substantial cytotoxicity in DU145, LNCaP, and 22Rv1 cells, which individually express either EGFR or HER3 (Figure 3, D and E, and Supplemental Figure 5, C–E). In contrast, PC-3 cells, characterized by low expression of both targets, exhibited the least sensitivity to BL-B01D1 (Figure 3, D and E, and Supplemental Figure 5, C–E). Conversely, SI-B001 showed no discernible cytotoxic activity in any of the tested cell lines (Figure 3D, and Supplemental Figure 5, C and D), suggesting that inhibition of EGFR/HER3 signaling alone is insufficient to impede prostate cancer cell proliferation, aligning with failures of previous clinical trials of EGF pathway inhibitors in advanced prostate cancer (12–14). Additionally, we observed that the isotype IgG ADC exerted cytotoxicity at high concentrations across multiple cell lines (Figure 3D, and Supplemental Figure 5C).

To further confirm the target-dependent cytotoxicity of BL-B01D1, we engineered PC-3 cells (which express low levels of both targets) to overexpress EGFR alone, HER3 alone, or both. Overexpression of either receptor substantially increased the sensitivity of PC-3 cells to BL-B01D1, with EGFR-overexpressing cells showing greater sensitivity than HER3-overexpressing cells (Figure 3F), reflecting the higher binding affinity of the ADC for EGFR (21). Furthermore, cells overexpressing both targets showed the highest sensitivity (Figure 3F). In parallel, we knocked down the corresponding target in DU145 (EGFR-high) and 22Rv1 (HER3-high) cells, which resulted in markedly reduced sensitivity to BL-B01D1, further confirming target dependence (Figure 3, G and H).

**Figure 4:**
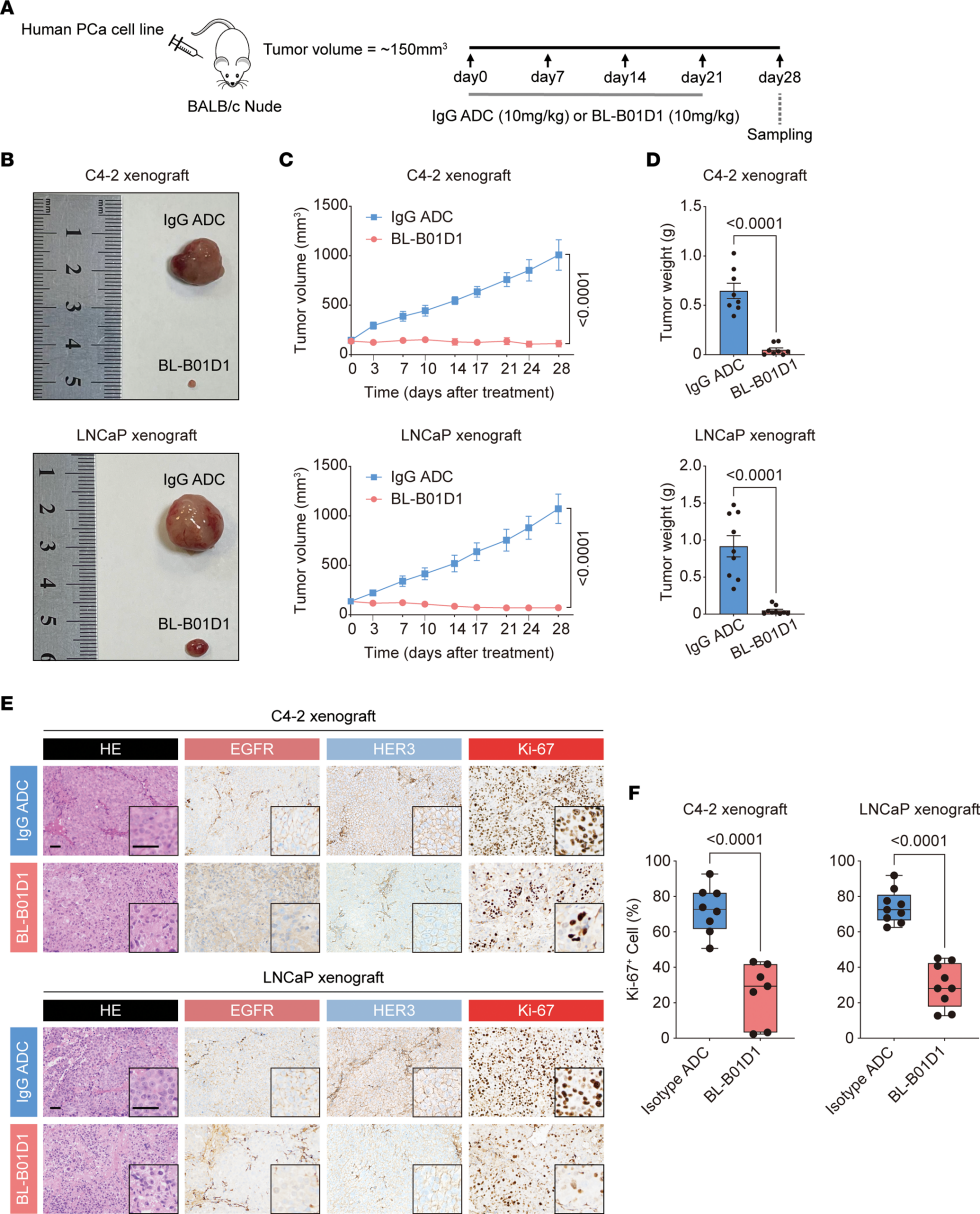
Antitumor activity of BL-B01D1 in human prostate cancer cell line models in vivo. (**A**) Xenograft study schematic. (**B**) Representative tumor images in C4-2 and LNCaP xenografts (*n* = 8/group for C4-2 xenograft; *n* = 9/group for LNCaP xenograft here and for remaining panels). (**C** and **D**) Tumor growth curves (**C**) and final tumor weights (**D**) in mice treated with BL-B01D1 or isotype IgG ADC. (**E**) Representative H&E and IHC staining of EGFR, HER3, and Ki-67 in C4-2 and LNCaP xenografts. Scale bar, 50 μm. (**F**) Quantification of Ki-67–positive tumor cells in C4-2 and LNCaP xenografts. Data are reported as mean ± SEM (**C** and **D**). The box plot represents the IQR divided by the median (**F**). Statistical significance was determined by 2-tailed unpaired *t* test (**C**, **D**, and **F**).

We then evaluated the in vivo antitumor activity of BL-B01D1 in prostate cancer xenograft models. Compared with the isotype IgG ADC, BL-B01D1 elicited robust antitumor effects in C4-2 and LNCaP tumor-bearing mice, with complete tumor regression observed in 1 of 8 C4-2 xenograft mice (Figure 4, A–D). Unexpectedly, BL-B01D1 also demonstrated marked antitumor activity in PC-3 xenografts (Supplemental Figure 6, A–D), which may be attributed to the relatively high dosing or potential influences from the tumor microenvironment (40). Throughout the treatment period, mouse body weights were monitored. Except for mice in the control group treated with isotype IgG ADC, which exhibited weight loss during the late phase due to tumor progression, BL-B01D1–treated mice maintained stable body weights relative to baseline, suggesting a favorable safety profile (Supplemental Figure 6, E-G). At the end of treatment, tumor tissues were collected for IHC analysis of target expression. EGFR and HER3 expression remained detectable in BL-B01D1–treated tumors, with a reduction in EGFR expression observed in C4-2 xenografts treated with BL-B01D1, where some tumor cells lost membranous EGFR staining and displayed predominantly cytoplasmic localization (Figure 4E, and Supplemental Figure 6, H and I). H&E staining revealed disorganized tumor architecture and features of cellular senescence (e.g., flattened morphology, vacuolization), and Ki-67 staining showed a marked reduction in proliferative cells, indicating substantial inhibition of tumor cell proliferation (Figure 4, E and F).

**Figure 5:**
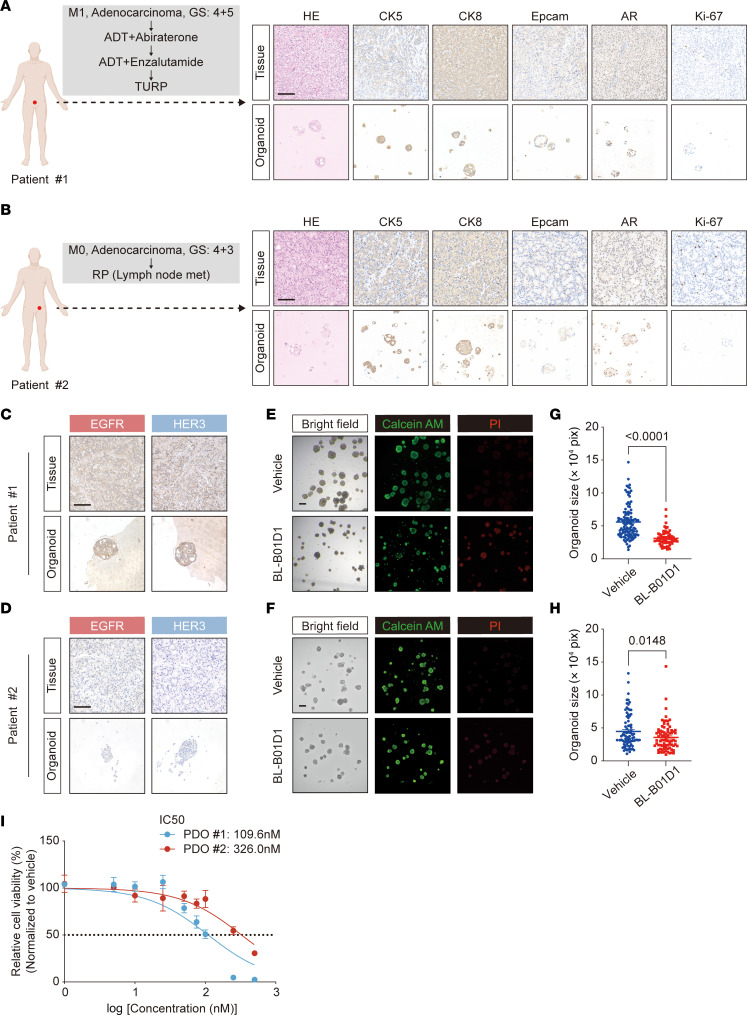
Antitumor activity of BL-B01D1 in PDO models. (**A** and **B**) Clinical history and histopathologic validation of 2 patients with prostate cancer from whom PDOs were established. H&E and IHC staining of original tumor tissues and corresponding PDOs are shown for CK5, CK8, EpCAM, AR, and Ki-67. Scale bar, 100 μm. GS, Gleason score; met, metastasis; RP, radical prostatectomy; TURP, transurethral resection prostate. (**C** and **D**) IHC staining of EGFR and HER3 in tumor tissues and matched PDOs. Scale bar, 100 μm. (**E** and **F**) Representative bright-field and fluorescence images of Calcein AM (green) and PI (red) staining of PDOs after treatment with BL-B01D1 (100 nM) or vehicle for 6 days (*n* = 3). Scale bar, 200 μm. (**G** and **H**) Quantification of organoid size following 6-day treatment with BL-B01D1 (100 nM) or vehicle treatment in PDO 1 (**G**) and PDO 2 (**H**), respectively. Pix, pixels. (**I**) Cell viability assay (CellTiter-Glo) of PDOs treated with increasing concentrations of BL-B01D1 for 6 days (*n* = 4). Data represent individual organoids (**G** and **H**) or mean ± SD (**I**). Statistical significance was determined by 2-tailed unpaired t test (**G** and **H**).

In addition to cell line-derived models, we also established PDOs from 2 patients with prostate cancer to further assess the therapeutic efficacy of BL-B01D1 (Figure 5, A and B). PDO models offer several advantages, including better preservation of intratumoral heterogeneity, native tissue architecture, and patient-specific molecular characteristics (41). One PDO originated from a tumor with high EGFR and HER3 expression; the other was derived from a tumor with low expression of both targets (Figure 5, C and D). Immunohistochemical analysis confirmed good concordance of EGFR and HER3 expression between the PDOs and their corresponding primary tumors (Figure 5, C and D). To functionally evaluate the therapeutic efficacy of BL-B01D1 in PDO models, we treated both PDOs with BL-B01D1 or vehicle control and assessed organoid viability using Calcein AM/propidium iodide (PI) live-dead staining, followed by high-content imaging. In the EGFR^high^/HER3^high^ PDO (PDO 1), BL-B01D1 treatment (100 nM) led to a marked reduction in viable organoid size and number, with a substantial increase in PI-positive (dead) organoids compared with vehicle control, indicating effective induction of cell death (Figure 5E). Quantitative analysis revealed a significant reduction in organoid size upon BL-B01D1 treatment (Figure 5G), consistent with potent cytotoxic activity. In contrast, the EGFR^low^/HER3^low^ PDO (PDO 2) showed more modest morphological changes and a less pronounced reduction in organoid viability following BL-B01D1 exposure (100 nM) (Figure 5F). Although a statistically significant decrease in organoid size was still observed, the magnitude of response was markedly lower than that seen in PDO 1 (average fold change relative to vehicle: 0.557 for PDO 1 vs. 0.796 for PDO 2) (Figure 5H). To further quantify drug response, we performed a CellTiter-Glo viability assay across a concentration gradient of BL-B01D1. The results revealed a substantially lower IC_50_ in the PDO 1 (109.6 nM) compared with the PDO 2 (326.0 nM) (Figure 5I).

In summary, these results suggest that BL-B01D1 exhibited potent and target-dependent antitumor activity across multiple prostate cancer models. Its efficacy correlated with EGFR and HER3 expression levels and was confirmed in both engineered cell lines and PDOs. These findings provide a strong rationale for further clinical development and highlight the utility of PDOs as translational models for preclinical drug evaluation.

### Clinical efficacy of BL-B01D1 in a patient with mCRPC.

**Figure 6:**
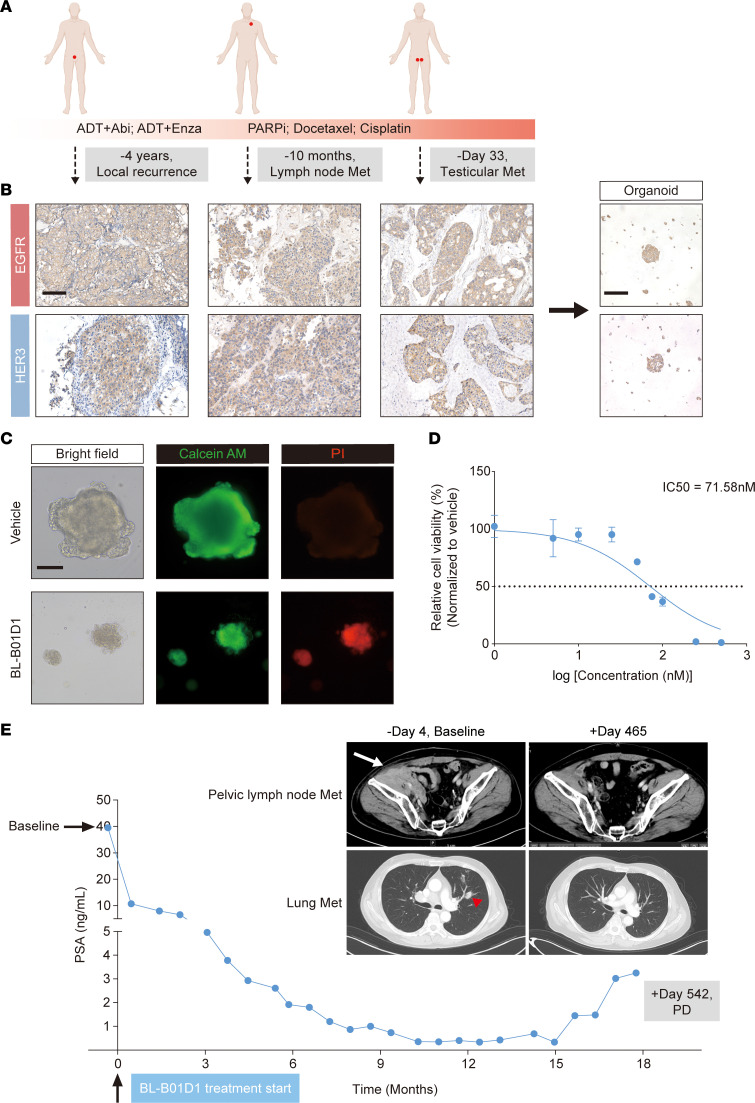
Clinical efficacy of BL-B01D1 in a patient with mCRPC and matched PDO validation of drug response. (**A**) Clinical course of the patient who received BL-B01D1 treatment, including treatment history and timeline of longitudinal sample collection. Abi, abiraterone; Enza, enzalutamide; Met, metastasis; PARPi, poly (ADP-ribose) polymerase inhibitor. (**B**) Longitudinal IHC analysis of EGFR and HER3 expression in locally recurrent lesion and metastatic lesions from the patient. IHC staining was also performed on the PDO generated from the testicular metastasis. Scale bar: 100 μm for tissue sections and PDO. (**C**) Representative bright-field and fluorescence images of Calcein AM (green) and PI (red) staining of PDO after treatment with BL-B01D1 (100 nM) or vehicle for 6 days (*n* = 3). Scale bar, 100 μm. (**D**) Cell viability assay (CellTiter-Glo) of the PDO treated with increasing concentrations of BL-B01D1 for 6 days (*n* = 4). Data are reported as mean ± SD. (**E**) PSA levels and representative radiographic images at baseline and during BL-B01D1 treatment. The white arrow indicates the pelvic lymph node metastasis at baseline; the red arrowhead marks the pulmonary metastatic lesion at baseline. PD, progressive disease.

To validate the clinical relevance of BL-B01D1 activity observed in preclinical prostate cancer models, we recommended a patient whose PDO exhibited marked sensitivity to BL-B01D1 for enrollment in an ongoing phase IIa/IIb trial of BL-B01D1 in patients with locally advanced or metastatic genitourinary tumors (ClinicalTrials.gov NCT05785039). The patient was diagnosed with prostate cancer at age 48 years, with an initial prostate-specific antigen (PSA) level of 10.25 ng/mL and underwent neoadjuvant ADT followed by salvage prostatectomy (T_4_N_1_M_1_; Gleason score: 5+4). A commercial genomic test revealed pathogenic mutations in *CDK12*. The patient subsequently underwent a series of standard therapies, including ADT, AR signaling inhibitors, PARP inhibitor, platinum-based and docetaxel chemotherapy, receiving a total of 9 lines of prior systemic treatment before BL-B01D1 trial enrollment (Figure 6A).

Before trial enrollment, bilateral testicular metastases were identified and histologically confirmed to originate from prostate cancer following orchiectomy. Immunohistochemical analysis of the metastatic lesions revealed high co-expression of EGFR and HER3 (Figure 6B). Additionally, we obtained FFPE samples from the patient’s neck lymph node metastasis taken 10 months prior to the initiation of BL-B01D1 therapy, as well as from the patient’s locally recurrent prostate cancer lesion 4 years before treatment. Immunohistochemical staining demonstrated consistent high expression of EGFR and HER3 in both the locally recurrent lesion and the neck lymph node metastasis (Figure 6B). A PDO model was successfully generated from the resected testicular metastasis, which retained elevated EGFR and HER3 expression (Figure 6B) and demonstrated marked sensitivity to BL-B01D1 (IC_50_ = 71.58 nM) (Figure 6, C and D). These findings supported the patient’s enrollment into the BL-B01D1 clinical study.

At the time of enrollment, the patient was 54 years old. He received BL-B01D1 at a dose of 2.5 mg/kg on days 1 and 8 every 3 weeks. After the first treatment cycle, his PSA level declined from 39.5 (baseline) to 10.7 ng/mL (Figure 6E). By the fourth cycle, radiographic assessment revealed partial responses in multiple target lesions (Figure 6E). The patient had a sustained response to BL-B01D1 and remained free of radiographic or clinical progression for 24 cycles of treatment, after which new-onset cervical lymph node metastasis was detected (Figure 6E).

### ABCG2 as a potential target to overcome BL-B01D1 resistance.

**Figure 7:**
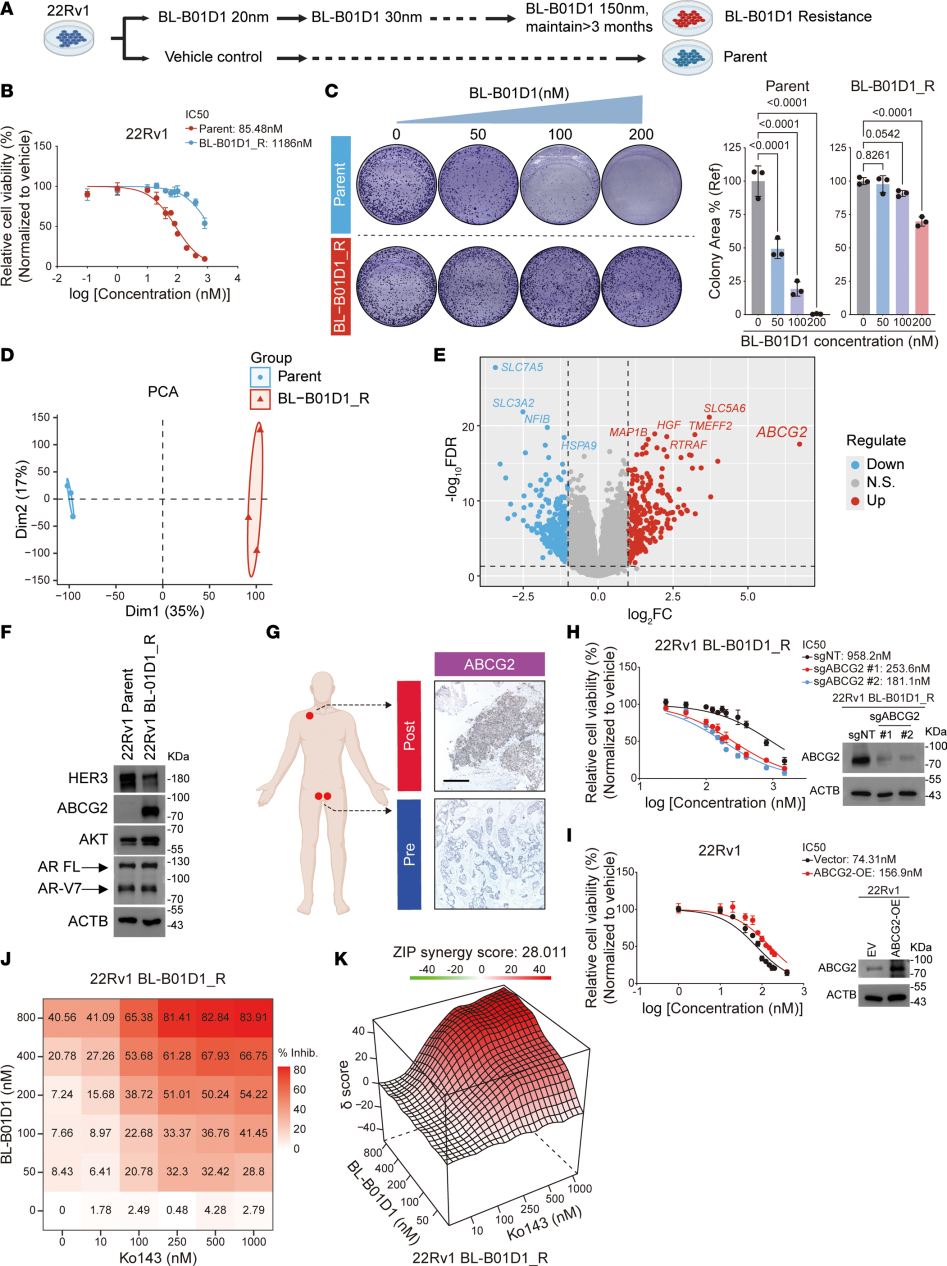
ABCG2 upregulation mediates acquired resistance to BL-B01D1. (**A**) Schematic of the generation of the 22Rv1 BL-B01D1_R subline. (**B**) Cell viability assays of parental and BL-B01D1_R cells treated with escalating doses of BL-B01D1 (*n* = 3). (**C**) Representative images and quantification of colony formation assays of parental and resistant cells treated with the indicated concentrations of BL-B01D1 (*n* = 3). (**D**) Principal component analysis (PCA) based on RNA-Seq data comparing transcriptional profiles of parental and resistant cells. (**E**) Volcano plot showing differentially expressed genes between parental and BL-B01D1_R cells. FC, fold change. (**F**) Immunoblot of the indicated proteins in parental and BL-B01D1–resistant 22Rv1 cells. (**G**) IHC staining of ABCG2 in pretreatment (Pre) and progressive lesions from the patient who developed resistance to BL-B01D1 treatment. Scale bar, 200 μm. Post, post-treatment. (**H**) Cell viability of 22Rv1-BL-B01D1_R/sgNT and 22Rv1-BL-B01D1_R/sgABCG2 treated with increasing concentration of BL-B01D1 (*n* = 3). Corresponding Western blot results are shown. (**I**) Cell viability of 22Rv1/vector and 22Rv1/ABCG2-OE treated with increasing concentration of BL-B01D1 (*n* = 3). Corresponding Western blot results are shown. (**J**) Dose-response matrix of 22Rv1 BL-B01D1_R cells after treatment with a combination of BL-B01D1 and Ko143 (*n* = 3). Matrix was generated by online SynergyFinder 3.0 software. % Inhib., percentage of inhibition. (**K**) Zero-interaction potency (ZIP) synergy score of BL-B01D1 in combination with Ko143 in 22Rv1 BL-B01D1_R cells (*n* = 3), calculated using the online SynergyFinder 3.0 software. Data are reported as mean ± SD (**B**, **C**, **H**, and **I**). Statistical significance was determined by 1-way ANOVA with Dunnett’s multiple comparisons (**C**).

With growing interest in understanding resistance to ADCs (42, 43), we sought to delineate the mechanisms contributing to BL-B01D1 resistance in prostate cancer cells. We established a BL-B01D1–resistant subline (22Rv1 BL-B01D1_R) by gradually exposing parental 22Rv1 to increasing concentrations of BL-B01D1 over several months (Figure 7A). The resistant subline showed a markedly elevated IC_50_ for BL-B01D1, indicating a substantial reduction in drug sensitivity compared with the parental cells (Figure 7B). Consistent with this phenotype, sensitivity to Ed-04, the cytotoxic payload of BL-B01D1, was also markedly reduced in the resistant cells (Supplemental Figure 7A). In colony formation assays, 22Rv1 BL-B01D1_R cells maintained proliferative capacity even at high concentrations of BL-B01D1 (100–200 nM), whereas parental cells showed almost complete suppression of colony growth under the same treatment conditions (Figure 7C).

To explore the molecular changes associated with resistance, we performed RNA-Seq on both parental and resistant cell lines. Principal component analysis revealed distinct transcriptional profiles between the 2 groups (Figure 7D). Differential expression analysis identified several genes that were upregulated in the resistant subline, with *ABCG2* showing the highest fold increase (Figure 7E). As a well-established ATP-binding cassette (ABC) transporter, ABCG2 has been implicated in drug resistance across various cancer types (44). Hierarchical clustering and heatmap visualization further confirmed the marked upregulation of *ABCG2* and other ABC transporters, such as *ABCC1* and *ABCC2*, in the resistant cells (Supplemental Figure 7B). Western blot analysis confirmed the upregulation of ABCG2 in 22Rv1 BL-B01D1_R cells (Figure 7F). Besides, HER3 expression was markedly downregulated in the resistant subline at both the protein and mRNA levels (Figure 7F and Supplemental Figure 7C), implying that reduced target engagement may contribute to the development of resistance. In contrast, AKT expression was modestly increased (Figure 7F), potentially reflecting adaptive activation of prosurvival signaling pathways. Meanwhile, the expression levels of full-length AR and AR-V7 remained largely unchanged, suggesting that the resistance phenotype was not mediated by alterations in canonical AR signaling (Figure 7F). Given that short-term blockade of HER3 reduced AR protein levels in parental 22Rv1 cells (Supplemental Figure 5B), this observation also suggests that during the development of resistance, the cells may adopt compensatory mechanisms to maintain AR stability despite sustained target inhibition. Importantly, analysis of the progressive cervical lymph node metastasis from the previously described representative patient revealed marked upregulation of ABCG2 compared with the baseline testicular metastasis, mirroring the molecular alterations observed in the resistant cell line (Figure 7G). Meanwhile, EGFR expression was reduced, whereas HER3 expression remained high (Supplemental Figure 7D).

To validate the role of ABCG2 in resistance, we knocked out, using CRISPR-Cas9, ABCG2 in the resistant cells. This resulted in a marked restoration of BL-B01D1 sensitivity, as evidenced by a substantial reduction in IC_50_ values from 958.2 nM in control cells (sgNT) to 253.6 nM and 181.1 nM in ABCG2 knockout cells (sgABCG2#1 and sgABCG2#2, respectively) (Figure 7H). Conversely, overexpressing ABCG2 in parental cells conferred resistance to BL-B01D1, as indicated by an increase in IC_50_ from 74.31 nM (vector) to 156.9 nM (ABCG2-OE), without substantially affecting cell proliferation in the absence of treatment (Figure 7I and Supplemental Figure 7E). To further validate this observation, we overexpressed ABCG2 in another prostate cancer cell line, C4-2. Consistent with the results in 22Rv1 cells, ABCG2 overexpression markedly increased resistance to BL-B01D1 and Ed-04 in C4-2 cells (Supplemental Figure 7, F and G), supporting the generalizability of this resistance mechanism in prostate cancer. Additionally, we observed that Ko143, a selective ABCG2 inhibitor, exhibited minimal cytotoxicity when administered alone to either parental or resistant cells (Supplemental Figure 7H). However, cotreatment with Ko143 and BL-B01D1 markedly resensitized the resistant cells to BL-B01D1 (Supplemental Figure 7I). The synergy index was calculated using the zero-interaction potency model (45), yielding a value of 28.011 and indicating a strong therapeutic synergy between Ko143 and BL-B01D1 in resistant cells (Figure 7, J and K). These findings suggest that ABCG2 plays a crucial role in mediating acquired resistance to BL-B01D1 in prostate cancer. Targeting ABCG2 may therefore provide a promising strategy to overcome resistance.

## Discussion

Several previous studies have demonstrated that the EGF signaling pathway plays a critical role in prostate cancer metastasis and resistance to endocrine therapies (6–11, 46). This has spurred clinical enthusiasm for exploring EGF-targeted therapies in patients with advanced prostate cancer (12–14). Although blockade of the EGF pathway has shown promising antitumor activity and the ability to reverse therapeutic resistance in several preclinical models of prostate cancer (10, 11, 47), EGF-targeted clinical trials have ultimately failed, indicating that blockade of the EGF pathway alone is not a feasible therapeutic strategy in advanced prostate cancer (12–14). ADC, as an emerging therapeutic modality, is transforming the landscape of cancer treatment and offers an opportunity to reevaluate previously unsuccessful therapeutic targets (15, 16). Previously, ADC target development in prostate cancer was mainly targeted at antigens with high and specific expression in prostate cancer cells, such as prostate-specific membrane antigen (PSMA) (48). In a phase II, single-arm trial involving 119 patients, only 14% of patients with mCRPC achieved a PSA response of at least a 50% decline from baseline after PSMA ADC treatment (49); another phase I/II trial reported an objective response rate of just 8%, with substantial peripheral neuropathy limiting the therapeutic window (50). These results suggest that PSMA-targeted ADCs may not be an optimal strategy, possibly due to marked heterogeneity in PSMA expression (51, 52), underscoring the need for alternative therapeutic targets for ADC development in prostate cancer.

Iza-bren is a first-in-class, bispecific ADC targeting EGFR and HER3 (21). This bispecific design synergistically broadens patient eligibility while enhancing internalization, effectively circumventing target heterogeneity (22, 23). In a phase I clinical trial involving 195 patients with advanced or metastatic solid tumors (mainly lung and nasopharyngeal cancers), BL-B01D1 demonstrated notable antitumor activity, with 34.5% of evaluable patients (*n* = 60 of 174) achieving an objective response and a median progression-free survival of 5.7 months (24). Among 40 patients with *EGFR*-mutant non–small-cell lung cancer, 52.5% (*n* = 21 of 40) experienced objective responses (24). Treatment-related toxicities were dominated by hematologic adverse events, which aligns with the camptothecin-derived topoisomerase-I payload (24, 53). ERBB-related mucocutaneous events such as stomatitis, diarrhea, and skin disorders (54, 55) were generally mild, indicating that the overall off-tumor effects associated with EGFR/HER3 engagement were clinically acceptable (24). Multiple phase III trials are ongoing to assess BL-B01D1 across various solid tumors, including lung, urothelial, and breast cancers (ClinicalTrials.gov identifiers NCT06500026, NCT06382129, NCT06838273, NCT06857175, NCT06382116, NCT06382142, NCT06118333, NCT06304974, NCT06343948, and NCT06926868).

Our multi-omics analysis revealed a marked increase in *EGFR* and *ERBB3* gene amplification events in advanced CRPC compared with localized prostate cancer, consistent with previous studies showing their upregulation contributes to disease progression and therapeutic resistance (6, 8–11). Protein expression analyses of CRPC specimens, integrated with transcriptomic data, demonstrated that although EGFR and HER3 were largely absent in CRPC-NE, approximately three-fourths of CRPC-Ad tumors expressed at least 1 of the 2 receptors, and about one-fifth co-expressed both, supporting their potential as therapeutic targets in CRPC-Ad and suggesting a complementary relationship. Notably, discrepancies between RNA and protein expression merit attention. For example, although *ERBB2* transcripts are frequently detected at relatively high levels and are comparable to highly expressed targets such as PSMA or TROP2, most prostate cancers are HER2-negative by clinical-grade IHC (33), possibly due to post-transcriptional or post-translational regulatory mechanisms. In contrast, EGFR and HER3 proteins were detectable in a substantial fraction of CRPC-Ad tumors, highlighting their feasibility as ADC targets. Preliminary analysis of paired HSPC and CRPC samples revealed a trend toward increased EGFR and HER3 expression in the CRPC stage. Although the sample size was limited, these findings suggest the possibility of dynamic changes in target expression during disease progression, which warrants further validation in larger cohorts.

Given the expression of EGFR and HER3 in a high proportion of prostate cancer, we tested the antitumor efficacy of BL-B01D1 in various preclinical prostate cancer models with different expression profiles. Our data showed that its parental antibody SI-B001 exhibited negligible antitumor effects in vitro, even at high concentrations, highlighting that EGF pathway blockade alone is insufficient to directly inhibit prostate cancer growth—consistent with the failure of compounds targeting this pathway in prior clinical trials (12–14). BL-B01D1 displayed dose-dependent cytotoxicity in multiple models, and its on-target effects were confirmed using overexpression and knockdown systems. Furthermore, in vivo studies showed that BL-B01D1 treatment reduced EGFR and HER3 expression in residual tumors, with a shift in localization from the cell membrane to the cytoplasm, suggesting that tumor cells may develop BL-B01D1 resistance through both downregulation of target expression and alteration of subcellular localization. Encouraged by its preclinical efficacy, we identified a heavily pretreated patient with mCRPC whose PDO exhibited marked in vitro sensitivity to BL-B01D1 and recommended the patient for enrollment in an ongoing phase II trial (ClinicalTrials.gov NCT05785039). The patient’s cancer subsequently demonstrated a clinical response concordant with the PDO. This finding highlights the potential of PDOs to serve as clinically relevant, patient-matched platforms for predicting drug response and resistance. Expanding the use of PDOs in future studies may facilitate biomarker-driven therapeutic stratification and accelerate the development of personalized ADC-based strategies in prostate cancer. Longitudinal analysis of specimens from this patient revealed modest changes in EGFR and HER3 expression levels over the course of treatment. These variations may reflect intrinsic differences between lesions from distinct anatomic sites or treatment-induced alterations in target expression. Further studies are warranted to better characterize the interlesional heterogeneity and dynamic changes in EGFR and HER3 expression during the clinical management of advanced prostate cancer.

Resistance to ADCs can arise through diverse mechanisms, broadly classified into target-related and payload-related pathways (56, 57). Target-related resistance includes downregulation, loss, or heterogeneous expression of target antigens, as well as changes in their cellular localization, impairing ADC binding or internalization. Payload-related resistance involves enhanced drug efflux or intrinsic insensitivity of tumor cells to the cytotoxic payload (58–60). We established BL-B01D1–resistant cell lines in vitro and, via transcriptomic comparisons with parental cells, identified *ABCG2* as the most significantly upregulated gene. ABC transporter family genes play a critical role in cancer drug resistance by actively exporting various anticancer agents out of the cell, thereby reducing intracellular drug concentrations and diminishing cytotoxic effects—a key factor in multidrug resistance (61). Representative members of this family, such as *ABCB1*, *ABCC1*, and *ABCG2*, have been widely implicated in anticancer drug resistance (62). Notably, in a patient treated with BL-B01D1, we observed a marked upregulation of ABCG2 in tumor specimens collected after the development of resistance compared with pretreatment samples, validating the clinical relevance of our in vitro findings. Cotreatment with the ABCG2 inhibitor Ko143 restored BL-B01D1 sensitivity in resistant cells, supporting ABCG2 as a functional mediator of acquired resistance. Although Ko143 itself is not clinically translatable, these findings highlight efflux-mediated resistance as a therapeutically actionable vulnerability. Based on the emerging concept of dual-payload ADCs (63), it may be possible to design ADCs that simultaneously deliver a conventional cytotoxic payload along with an agent that mitigates transporter-mediated efflux, providing a potential strategy to overcome resistance.

This study has several limitations. First, we assessed the protein expression of EGFR and HER3 primarily in patients with CRPC-stage disease from a single center. Due to the limited availability of paired HSPC and CRPC specimens, the number of HSPC samples was small, and the expression profiles of these 2 targets at the HSPC stage remain largely unknown. Moreover, due to the limited accessibility of metastatic lesion biopsy specimens from patients with advanced disease, assessment of interlesional and temporal heterogeneity in the expression of these targets was feasible in only a small subset of patients. In addition, due to the inherent fragility of prostate cancer cells, we were only able to establish a single BL-B01D1–resistant cell line, and the investigation of resistance mechanisms was confined to in vitro models. Furthermore, knockdown efficiency was relatively low in some cell lines, which may have attenuated the magnitude of IC_50_ shifts observed in vitro. Although our study demonstrates that BL-B01D1 exhibits antitumor activity in PC-3 xenografts despite low EGFR/HER3 expression, the contribution of tumor microenvironmental factors to this observation was not specifically investigated.

In conclusion, this study provides translational evidence supporting EGFR and HER3 as viable targets in mCRPC and demonstrates that the bispecific ADC BL-B01D1 exerts potent, target-dependent antitumor activity across multiple models. The encouraging preliminary clinical response in a heavily pretreated patient with mCRPC together with matched organoid validation and mechanistic insights into resistance, support the continued clinical development of BL-B01D1. In light of these results, the phase IIa/IIb clinical trial (ClinicalTrials.gov NCT05785039) has been expanded to incorporate an mCRPC arm, which is currently ongoing.

## Methods

### Sex as a biological variable.

Our study exclusively examined male mice because the disease modeled is only relevant in the male sex.

### Patients and samples.

We obtained FFPE tumor specimens from 70 patients with CRPC who were treated at FUSCC between March 2016 and April 2024, including 16 patients exhibiting neuroendocrine differentiation (CRPC-NE). CRPC-NE was defined by the presence of immunohistochemical staining for CD56, chromogranin, or synaptophysin in more than 20% of tumor cells (36, 64). In addition, matched FFPE samples from the hormone-sensitive stage, including prostate biopsy specimens and samples from patients who underwent radical prostatectomy, were available for 10 of these patients. Also, a pelvic lymph node metastasis from a patient with hormone-sensitive prostate cancer, transurethral resection of the prostate tissue from a patient with CRPC, and bilateral testicular metastases from another patient with CRPC were collected to establish PDOs. Pathology was reviewed by a certified pathologist (H.G.) with expertise in prostate cancer pathology, who was blinded to clinical data. Clinical data for each patient were retrospectively collected from the hospital electronic patient record system. We collected the clinical characteristics and data on response to BL-B01D1 of a patient enrolled in an ongoing clinical trial (ClinicalTrials.gov NCT05785039). Details of the clinical trial protocol, including the study design, eligibility criteria, and treatment regimen, were described in our report of our previous study (65). To investigate EGFR and HER3 dynamics during disease progression and treatment, we also collected additional longitudinal FFPE specimens from this patient at different treatment stages, including 2 distinct distant lymph node metastases and a locally recurrent prostate cancer lesion.

### Cell lines.

The human prostate cancer cell lines C4-2 (RRID: CVCL_4782) and LNCaP (RRID: CVCL_0395) were purchased from the American Type Culture Collection. The lines 22Rv1 (RRID: CVCL_1045), DU145 (RRID: CVCL_0105), PC-3 (RRID: CVCL_0035), and HEK293T (RRID: CVCL_0063) were purchased from Cell Bank (Shanghai Institutes for Biological Sciences, Chinese Academy of Sciences). LNCaP, C4-2, 22Rv1 and PC-3 were cultured in RPMI1640 medium (Gibco, C11875500BT), and DU145 and HEK293T were cultured in DMEM (Gibco, C11995500BT). The base medium was supplemented with 10% FBS (Gibco, A5256701) and a 1% penicillin-streptomycin solution (Meilunbio, MA0110). All cells were kept at 37°C in a humidified incubator with 5% CO_2_. All cells were authenticated by short tandem repeat analysis and confirmed negative for *Mycoplasma* contamination by monthly testing with the Mycoplasma Detection Kit (Yeasen, 40612ES60) according to the manufacturer’s protocol.

### IHC.

Paraffin-embedded tissue sections were deparaffinized, rehydrated, and subjected to antigen retrieval using EDTA buffer (pH 9.0; Beyotime, P0084). Endogenous peroxidase activity was blocked with 3% hydrogen peroxide. Sections were then blocked with 1% BSA and incubated with primary antibodies overnight at 4 °C. After incubation with a universal HRP-conjugated secondary antibody (Abclonal, RK50015), DAB staining (Servicebio, G1212-200T) was used for color development. Slides were counterstained with hematoxylin, dehydrated, and mounted for microscopic analysis. Images were acquired using an Olympus IXplore SpinSR microscope or a Leica DMi8 microscope. EGFR and HER3 expression for each sample was semi-quantitatively assessed by a certified pathologist (H.G.) blinded to clinical data, using H-scores that were calculated as follows: (% of weak staining × 1) + (% of moderate staining × 2) + (% of strong staining × 3) (66, 67). An H-score of 200 or higher was defined as high expression, and an H-score less than 200 was considered low expression (68). The proportion of Ki-67–positive tumor cells was quantified using the Trainable Weka Segmentation plugin implemented in Fiji (ImageJ version 1.54h, an ImageJ distribution; RRID: SCR_002285). The antibodies used are listed in Supplemental Table 2.

### Plasmids and lentiviral transfection.

The shRNA constructs were designed using the BLOCK-iT RNAi Designer (Thermo Fisher Scientific) and inserted into the pLKO.1 vector (RRID: Addgene_10878). For CRISPR/Cas9-mediated gene knockout, sgRNAs targeting *ABCG2* were designed using the CRISPOR (https://crispor.gi.ucsc.edu/) and cloned into the LentiCRISPR v2-GFP vector (RRID: Addgene_82416). The sequences for shRNA and sgRNA used in this study are summarized in Supplemental Table 3. The EGFR overexpression plasmid (pCDH-Flag-EGFR-Puro) and the corresponding empty vector were provided by Tongjin Zhao, Shanghai Key Laboratory of Metabolic Remodeling and Health, Institute of Metabolism and Integrative Biology, Zhongshan Hospital, Fudan University (69). To generate the HER3 overexpression plasmid, the coding sequence of *ERBB3* was amplified by PCR from a *ERBB3* template plasmid (BRICS, SP-105261) and subsequently cloned into the pLVX-IRES-Hygro (RRID: Addgene_164592). To generate the ABCG2 overexpression plasmid, the coding sequence of *ABCG2* was cloned from a cDNA library prepared from 22Rv1 BL-B01D1_R cells and subsequently cloned into the pCDH-EF1-copGFP-T2A-Puro (RRID: Addgene_72263).

For lentiviral packaging, the transfer plasmid and packaging plasmids (pMD2.G [RRID: Addgene_12259] and psPAX2 [RRID: Addgene_12260]) were cotransfected into 293T cells grown to approximately 70% confluence in 6-cm dishes using polyethyleneimine as the transfection reagent, at a mass ratio of 5:1:2.5. The culture medium was replaced 10 hours after transfection. Viral supernatants were collected at 48 and 72 hours after transfection. For transduction, prostate cancer cells at approximately 50% confluence were incubated with the viral supernatant in the presence of 8 μg/mL polybrene (Sigma, TR-1003-G). After 48 hours of infection, cells were subjected to antibiotic selection with puromycin (1 μg/mL; Meilunbio, MA0318) or hygromycin (100 μg/mL; Meilunbio, MA0210) for 7 days, or GFP-positive cells were enriched by FACS, depending on the construct used.

### Quantitative RT-PCR.

Total RNA was extracted from cultured cell lines, using the EZ-10 DNAaway RNA Mini Preps Kit (Sangon Biotech, B618133) according to the manufacturer’s instructions. cDNA was synthesized from the isolated RNA using the PrimeScript RT Master Mix (Takara, RR036B) following the manufacturer’s protocol. Quantitative RT-PCR was performed using the ChamQ Universal SYBR qPCR Master Mix (Vazyme, Q711-02), in accordance with the manufacturer’s instructions, on a LightCycler 480 Instrument II (Roche). Primer sequences used in this study are listed in Supplemental Table 4.

### Prostate cancer organoid culture and functional assays.

Primary prostate cancer tissues were dissected into 1–2 mm^3^ fragments, followed by sequential enzymatic digestion with Collagenase II (Solarbio, C8150) and TrypLE (Gibco, 12605-010) to generate single-cell suspensions. Epcam-positive cells were sorted (RRID: AB_2650909; Biolegend, 369813) by flow cytometry (Beckman; MoFloAstrios EQ), embedded in Matrigel (D1Med; D23016-0010) at a density of 1,000 cells/μL, and cultured in prostate cancer organoid medium supplemented with Y-27632 (ROCK inhibitor, GLPBIO, GC10512) for 3D growth (41, 70). Medium was refreshed every 2–3 days, and Y-27632 was omitted after 1 week. Key medium components are summarized in Supplemental Table 5.

For cryopreservation, organoids were isolated from Matrigel, centrifuged, and resuspended in CellBanker at a concentration of 1,000 organoids/mL, then stored in liquid nitrogen. Upon revival, frozen organoids were rapidly thawed in a 37°C water bath, washed with organoid revival solution (D1Med, D23040-0100), and replated in Matrigel, as described for Epcam-positive cells. For drug testing, organoids with diameters of 70–100 μm were treated with compounds, with medium replaced every 72 hours. On day 6, organoid morphology was assessed by bright-field imaging and quantified based on cross-sectional area (71). Cell viability was evaluated using the CellTiter-Glo 3D Cell Viability Assay (Promega, G9683) (72), and live/dead cell discrimination was performed using Calcein-AM (Yeasen, 40719ES50) and PI (Thermo, P3566) staining according to established protocols (73). The IC_50_ calculation for organoids mirrored the method used for cellular IC_50_ determinations.

### Animal experiments.

Male athymic nude BALB/c mice (RRID: IMSR_JAX:002019, The Jackson Laboratory), 6 weeks old, were housed in the Laboratory Animal Center of FUSCC under specific pathogen–free conditions with free access to food and water. Environmental parameters were controlled with a temperature of 22°C ± 0.5°C, humidity of 60% ± 3%, and a 12-hour light/12-hour dark cycle. After adaptive feeding for 1 week, all mice were enrolled in experiments. For in vivo treatment with BL-B01D1 or isotype IgG ADC, approximately 5 × 10^6^ LNCaP, C4-2, or PC-3 cells were subcutaneously injected into the flanks of male nude BALB/c mice. When tumors reached approximately 150 mm³, mice were randomized into 2 groups (*n* ≥ 6/group) with comparable average tumor volumes. Mice received either BL-B01D1 or isotype IgG ADC (10 mg/kg, i.v., once weekly). Tumor size was measured every 3–4 days using calipers, and tumor volume was calculated using the formula (length × width²)/2, in a blinded manner.

### Bulk RNA-Seq and data analysis.

RNA-Seq was performed on 22Rv1 cells treated with vehicle or resistant to BL-B01D1, with 3 duplicates of each group (Novogene). Briefly, total RNA was extracted using TRIzol reagent (Invitrogen, 15596026). RNA integrity was assessed using the Bioanalyzer 2100 system (Agilent Technologies). mRNA was purified from total RNA, using oligo-dT, and served as templates for cDNA synthesis. Sequencing was performed on the Illumina NovaSeq 6000 (Illumina). Transcriptome reads were mapped to the reference genome (hg38; https://www.ncbi.nlm nih.gov/assembly/GCF_000001405.26) using HISAT2 (version 2.0.5; RRID: SCR_015530). Gene expression levels were quantified by featureCounts (version 1.5.0-p3; RRID: SCR_012919), and fragments per kilobase of transcript per million mapped reads values were subsequently calculated based on gene lengths and library sizes. R (version 4.3.1; RRID: SCR_001905) and limma (version 3.58.1; RRID: SCR_010943) were used to perform data quality control and differential gene analysis. Genes with a FDR of less than 0.05 and an absolute log_2_ fold change greater than 1 were considered significantly differentially expressed.

### Analyses of published datasets.

Genomic, transcriptomic, and clinical data, as well as NEPC and AR signature scores from the TCGA–Prostate Adenocarcinoma (TCGA-PRAD) cohort (TCGA, Firehose Legacy), SU2C/Prostate Cancer Foundation (SU2C/PCF) Dream Team cohort (28), FHCRC cohort (29), and Weill Cornell Medicine cohort (36) were obtained from cBioPortal (http://www.cbioportal.org/). Genomic, transcriptomic, and clinical data from the CPGEA cohort (27) were downloaded from the National Genomics Data Center (https://ngdc.cncb.ac.cn/gsa-human/) under accession PRJCA001124. For CNA analysis, inferred copy number scores of 1 and 2 were defined as gain and amplification, respectively, in the TCGA, SU2C, and FHCRC cohorts. For the CPGEA cohort, CNA status was determined using Genomic Identification of Significant Targets in Cancer (GISTIC), version 2.0, scores (74), with a score of 1 defined as gain and of 2 as amplification. To evaluate the relative expression of EGF family genes, we performed a log_2_(*x* + 1) transformation on RNA-Seq expression values from the 4 datasets prior to ranking. For the comparison of *EGFR* and *ERBB3* mRNA expression between CRPC-Ad and CRPC-NE samples, we excluded specimens with unknown histological subtype from the SU2C/PCF Dream Team cohort. mRNA expression data for AR knockdown and control groups in the CWR22Pc prostate cancer cell line were downloaded from the GEO database (accession GSE68993) and normalized using the DESeq2 package (version 1.42.1; RRID: SCR_015687). Single-cell RNA-Seq data from 13 human prostate cancer samples were downloaded from the GEO database (accession GSE141445). Quality control was performed according to prior work (34). The resolution parameter for preclustering was set to 0.05, and cell type annotations were based on marker genes in prior studies (34, 75–77). Mouse single-cell RNA-Seq data were obtained from the NGDC database (https://ngdc.cncb.ac.cn/; accession OMIX001928). After normalization, using the “NormalizeData” function in the Seurat package (version 4.4.0; RRID: SCR_016341), batch effects were corrected using the “IntegrateData” function based on anchors identified by the “FindIntegrationAnchors” function. The resolution parameter for preclustering was set to 0.1. Clusters with high mitochondrial content were excluded. Cell-type annotation marker genes were assigned based on prior work (38).

To further examine EGFR and HER3 expression in luminal and neuroendocrine cells, we extracted these cell populations, excluding those derived from wild-type prostate tissue or with fewer than 2,000 detected RNA features. Tumor epithelial cells were reclustered using the “FindClusters” function with a resolution of 0.06, resulting in 2 distinct clusters. To assess AR, H3K27ac, and H3K4me2 binding at the *EGFR* and *ERBB3* loci, ChIP-Seq datasets from prostate cancer cell lines and tissues were downloaded from GEO (accession GSE161167, GSE96652, and GSE148935) and visualized using IGV (version 2.19.4; RRID: SCR_011793).

### Statistics.

Data are presented as mean ± SEM or mean ± SD, as indicated in the figure legends. Correlation analyses were performed using Pearson’s correlation coefficient. Statistical comparisons were conducted using 1-way ANOVA, 2-tailed Student’s *t* test, Wilcoxon signed-rank test, Mann-Whitney *U* test, or the Kruskal-Wallis *H* test, as appropriate. For multiple group comparisons, post hoc pairwise tests with appropriate multiple comparison corrections were applied. For in vivo studies, mice in the BL-B01D1 and isotype IgG ADC groups were randomized based on tumor burden prior to treatment initiation. Statistical methods used for all analyses are detailed in the corresponding figure legends. All analyses were performed using R (version 4.3.1; RRID: SCR_001905) or GraphPad Prism (version 10.1.2; RRID: SCR_002798). A *P* value <0.05 was considered statistically significant.

### Study approval.

All experiments involving human specimens were reviewed and approved by the FUSCC Ethics Committee (approval 050432-4-2307E, 2501-Exp132). Written informed consent was obtained from all participants. All animal experiments were approved by the FUSCC IACUC (FUSCC-IACUC-2024399, FUSCC-IACUC-2024523).

### Data availability.

RNA-Seq data generated during this study have been deposited in the GEO under accession code GSE301648. Values for all data points shown in the graphs are provided in the Supporting Data Values file. This study did not generate any unique code. All software and algorithms used in the study are freely or commercially available and are listed in Methods.

## Author contributions

BF, Yao Zhu, GW, DY, and XB designed the study. BF, XL, WM, YL, QL, and TZ performed the experiments. BF, HG, and ZW analyzed the data. XL, BF, and BW collected the patient samples. YL, Yi Zhu, and HZ provided technical support. BF and XL wrote the manuscript. Yao Zhu, GW, XB, and DY supervised the work and revised the manuscript. Yao Zhu acquired funding for the work. Authorship order among co–first authors was determined based on the relative extent of their contributions to the project.

## Conflict of interest

YZ and HZ are employed by Sichuan Biokin Pharmaceutical Co., Ltd. SX is employed by Baili-Bio (Chengdu) Pharmaceutical Co., Ltd.

## Funding support

- Natural Science Foundation of China (grants 82473381, 82172621, and 81972375 to Yao Zhu).
- Shanghai Medical Innovation Research Special Project (grant 21Y11904300 to Yao Zhu).
- General Program of Beijing Xisike Clinical Oncology Research Foundation (grant Y-MSDZD2021-0230 to Yao Zhu).
- Special Program for Post-marketing Clinical Research of Innovative Drugs (grant WKZX2023XC10001 to Yao Zhu).
- Program for Professor of Special Appointment (Easter Scholar) (grant TP2022051 to Yao Zhu).
- Beijing Weikang Prostate Cancer Research Special Fund (grant WK2024-003 to Yao Zhu).
- Shanghai Shenkang Research Physician Innovation and Transformation Ability Training Project (grant SHDC2022CRD035 to Yao Zhu).

## Supplementary Material

Supplemental data

Unedited blot and gel images

Supporting data values

## Version 1 04/07/2026

In-Press Preview

## Version 2 06/01/2026

Electronic publication
