# Expression of Concern for EGFR- and HER3-targeted bispecific antibody-drug conjugate demonstrates antitumor activity in metastatic castration-resistant prostate cancer

**DOI:** 10.1172/JCI213418

**Published:** 2026-10-01

**Authors:** Bangwei Fang, Xiaomeng Li, Ying Lu, Weiwei Ma, Hualei Gan, Tingwei Zhang, Qi Liu, Beihe Wang, Zixian Wang, Yi Zhu, Hai Zhu, Sa Xiao, Xiaojie Bian, Gonghong Wei, Dingwei Ye, Yao Zhu

Original citation: *J Clin Invest.* 2026;136(11):e201090. https://doi.org/10.1172/JCI201090

Citation for this expression of concern: *J Clin Invest.* 2026;136(19):e213418. https://doi.org/10.1172/JCI213418

The authors recently alerted the Editors to errors in the source data for figures 3D, S5D, and S7F that were identified in response to an institutional mandate for a post-publication audit of the data. Although the authors have indicated that the errors were due to inadvertent mistakes, the Editors are issuing this Expression of Concern to alert readers to the issues. We have requested institutional oversight into this matter and will alert readers to the outcome when the institutional evaluation is complete.

## Version 1 10/01/2026

Electronic publication

