# Supplementary material for: EGFR- and HER3-targeted bispecific antibody-drug conjugate demonstrates antitumor activity in metastatic castration-resistant prostate cancer
Source: J Clin Invest. 2026 Apr 7;136(11):e201090. doi: 10.1172/JCI201090 (PMC13221231; doi:10.1172/JCI201090)
Supplement: Supplemental data [file jci-136-201090-s051.pdf]

Supplemental information for

EGFR- and HER3-targeted bispecific antibody-drug conjugate demonstrates anti-tumor activity in metastatic castration-resistant prostate cancer

Bangwei Fang, Xiaomeng Li, Ying Lu, Weiwei Ma, Hualei Gan, Tingwei Zhang, Qi Liu, Beihe Wang, Zixian Wang, Yi Zhu, Hai Zhu, Sa Xiao, Xiaojie Bian, Gonghong Wei, Dingwei Ye, Yao Zhu

This Supplemental information contains:

Supplemental Methods

Supplemental Figures: 7

Supplemental Tables: 5

SUPPLEMENTAL METHODS

Long-term clonogenic assays

Prostate cancer cells were seeded in 6-well plates at a density of 5,000–10,000 cells per well, depending on growth rate. After 24 hours, cells were treated with vehicle or BL-B01D1 at the indicated concentrations and cultured for 9–14 days, with medium refreshed every 4 days. For combination assays with BL-B01D1 and Ko143, BL-B01D1-resistant cells were seeded in 12-well plates at 3,000 cells per well. After 24 hours, cells were treated with vehicle or the indicated drugs and cultured for 12 days, with medium changed every 4 days. Cells were fixed with 4% paraformaldehyde and stained with 0.1% crystal violet. Colony area was quantified using ImageJ (version 1.54h, RRID: SCR_003070).

siRNA transfection

The siRNA against EGFR, ERBB3, and the corresponding control RNA (siNC) were synthesized by GenePharma. C4-2 cells were seeded in 6-cm culture dishes at approximately 60% confluence. After 24 hours, Lipofectamine RNAiMAX reagent (Invitrogen) was diluted in Opti-MEM, and siRNAs were separately diluted in Opti-MEM according to the manufacturer's instructions. The diluted siRNAs were then mixed with the Lipofectamine RNAiMAX solution and incubated at room temperature for 15 min to allow complex formation. Subsequently, the siRNA–lipid complexes were added to the cells. The culture medium was replaced 24 hours after transfection, and cells were harvested 72 hours post-transfection. The sequences of the siRNAs are provided in Supplemental Table 3.

Western blot

Cells were harvested and lysed with RIPA buffer (Beyotime, #P0013B)

containing 1X protease (Bimake, #B14002) and phosphatase inhibitor cocktail (Beyotime, #P1081). BCA assay (Beyotime, #P0011) was used to measure protein concentration. Equivalent amounts of each sample were loaded on 8%-12% Bis-Tris gels, and proteins were transferred onto PVDF membranes (Millipore, #IPVH00010). The membrane was blocked with 5% skim milk diluted in 1X TBST for 2 hours at room temperature and incubated with primary antibodies overnight at 4°C. The next day, the membrane was washed three times with 1X TBST followed by incubation with secondary antibodies for 1 hour at room temperature. To assess the inhibitory effects of BL-B01D1 and its unconjugated backbone SI-B001 on EGFR/HER3 downstream signaling, prostate cancer cells were treated with vehicle, BL-B01D1, SI-B001, or isotype control ADC at a final concentration of 50 nmol/L for 24 hours. Following treatment, cells were either left unstimulated (PBS), or stimulated with recombinant human epidermal growth factor (EGF; MCE, #HY-P7109, 100 ng/mL for 10 minutes) or neuregulin-1 (NRG-1; MCE, #HY-P71171, 100 ng/mL for 20 minutes) prior to harvest. The antibodies used are summarized in Supplemental Table 2.

Flow cytometry

Prostate cancer cells were first harvested and resuspended in flow cytometry staining buffer (PBS with 2% FBS and 0.09% sodium azide) with approximately 1×10^6 cells per centrifuge tube. Human TruStain FcX™ (Biolegend, #422301, RRID: AB_2818986) was added to block Fc receptors, and the cells were incubated at room temperature for 10 minutes. Fluorochrome-conjugated antibodies against EGFR (Biolegend, #352904, RRID: AB_10896794) or HER3 (Biolegend, #324707, RRID: AB_2099568), or their respective isotype control antibodies, were added to separate samples, followed by incubation for 30 minutes at 4 °C in the dark. After staining, cells were washed twice with flow cytometry staining buffer and subsequently analyzed using a flow cytometer

(Thermo, Attune NxT).

Cell viability and IC50 determination

Cell viability and IC50 values were assessed using the CCK-8 assay (Meilun, #MA0218) for LNCaP, C4-2, PC-3 and DU145 cells, and the MTT assay (Beyotime, #ST316) for 22Rv1 cells. Prostate cancer cells (3,000–5,000 cells per well, depending on growth rate) were seeded in 96-well plates and incubated under standard culture conditions for 24 hours. Cells were then treated with various concentrations of drugs, with medium refreshed every 3 days. BL-B01D1, SIB001, and isotype IgG ADC (provided by SystImmune, Sichuan, China) were dissolved in PBS (Meilun, #MA0015), while Ko143 (MCE, #HY-10010) and Ed-04 (provided by SystImmune, Sichuan, China) were dissolved in DMSO (Sigma, #D8418). Cell viability was measured at specified time points—day 6 for BL-B01D1, SIB001, and isotype IgG ADC treatments, and day 4 for Ko143 and Ed-04. For combination studies, cells were treated with BL-B01D1 in the presence or absence of Ko143 using the same dosing schedule as BL-B01D1 monotherapy, and cell viability was assessed on day 6. For the CCK-8 assay, 10 µL of reagent was added to each well, followed by incubation at 37 °C for 1 hour. For 22Rv1/22Rv1_BL-B01D1_R cells, viability was assessed using the MTT assay by adding 0.5 mg/mL MTT and incubating at 37 °C for 4 hours. Absorbance was measured at 450 nm (for CCK-8) or 490 nm (for MTT) using a microplate reader. Cell viability was calculated using the following formula: (OD treatment – OD blank) / (OD control – OD blank) × 100%, where OD treatment, OD control, and OD blank represent absorbance readings from drug-treated, vehicle-treated, and media-only wells, respectively. IC50 values were determined using non-linear regression fitting of drug concentrations and relative cell viability in GraphPad Prism (version 10.1.2, RRID: SCR_002798). Drug synergy index was calculated using the SynergyFinder software (https://synergyfinder.fimm.fi) based on the zero-

interaction potency (ZIP) model (1).

SUPPLEMENTAL FIGURES

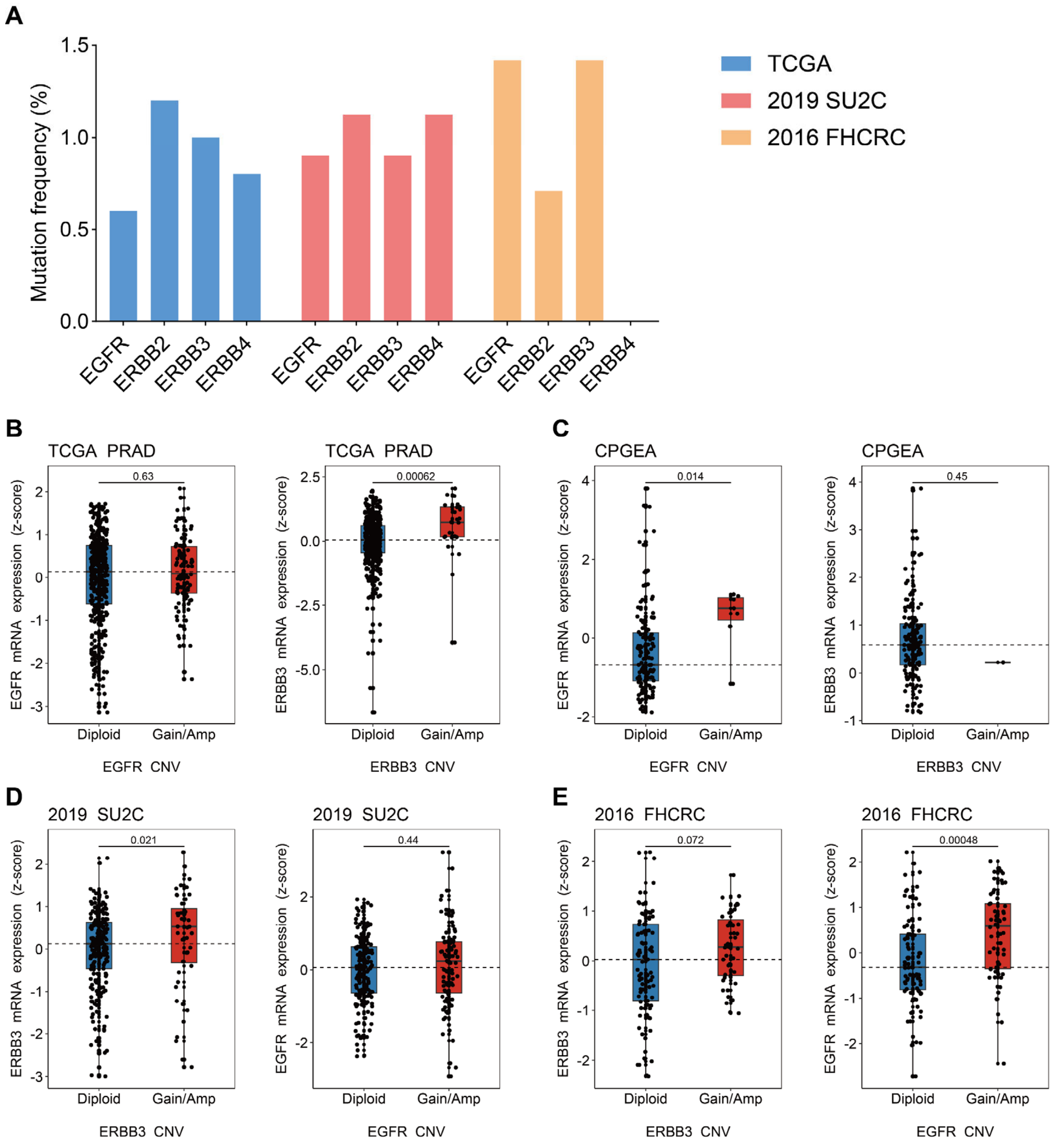

Supplemental Figure 1: Gene mutation frequency of EGF receptor family members and mRNA expression of *EGFR* and *ERBB3* in prostate cancer.

(A) Frequency of mutations in *EGFR*, *ERBB2*, *ERBB3*, and *ERBB4* across localized prostate cancer (TCGA, n = 492) and mCRPC cohorts (SU2C, n = 444; FHCRC, n = 149). Data from the CPGEA cohort (n = 208) are not shown, as no mutations in these four genes were detected in that dataset.

(B–E) mRNA expression levels of *EGFR* and *ERBB3* in prostate cancer samples from TCGA (B), CPGEA (C), SU2C (D), and FHCRC (E) cohorts, stratified by gene copy number status (Gain/Amplification vs. Diploid). Statistical significance was determined by Mann-Whitney U test.

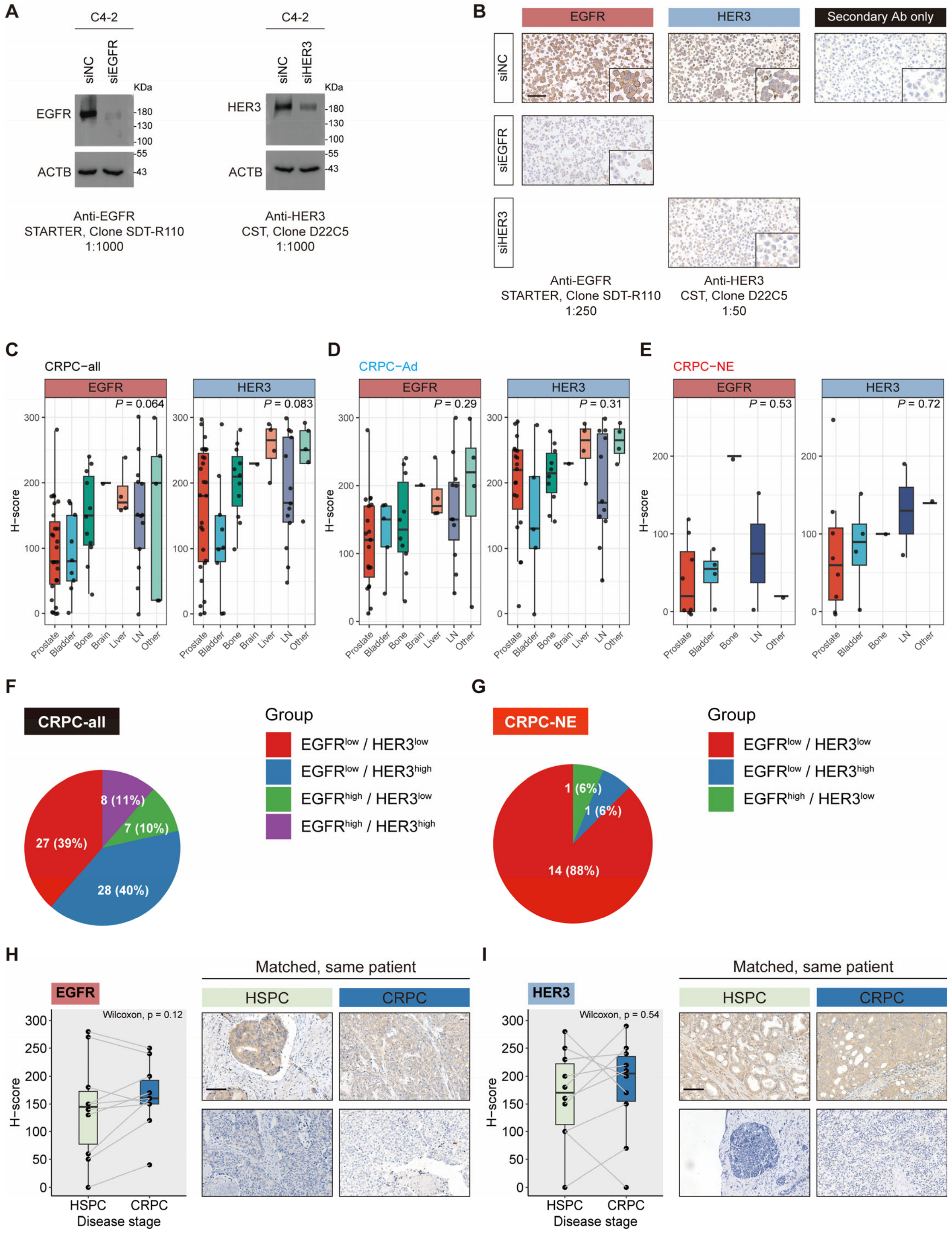

Supplemental Figure 2: EGFR and HER3 expression in CRPC specimens from various anatomical sites and matched HSPC–CRPC samples.

(A) Antibody validation by Western blot showing siRNA-mediated knockdown and non-targeting control siRNA. Left: anti-EGFR (clone SDT-R110, STARTER); right: anti-HER3 (clone D22C5, Cell Signaling Technology).

(B) Antibody validation by IHC showing siRNA-mediated knockdown and non-targeting control siRNA. Representative micrographs of FFPE cell pellet sections from the same experiment are shown, including secondary antibody–only controls. Scale bar, 100 µm.

(C–E) H-score quantification of EGFR and HER3 IHC staining across different metastatic sites in (C) all CRPC cases, (D) CRPC-Ad, and (E) CRPC-NE subtypes. The box plot represents the IQR divided by the median. Statistical significance was determined by Kruskal-Wallis H test.

(F–G) Pie charts showing EGFR/HER3 expression patterns in (F) all CRPC cases (n = 70) and (G) CRPC-NE (n = 16) tumor samples.

(H–I) Longitudinal IHC analysis of matched HSPC and CRPC tumor samples (n = 10 patients), showing (H) EGFR and (I) HER3 expression changes. The box plot represents the IQR divided by the median. Statistical significance was determined by Wilcoxon signed-rank test. Representative IHC images are shown on the right. Scale bar, 100 µm.

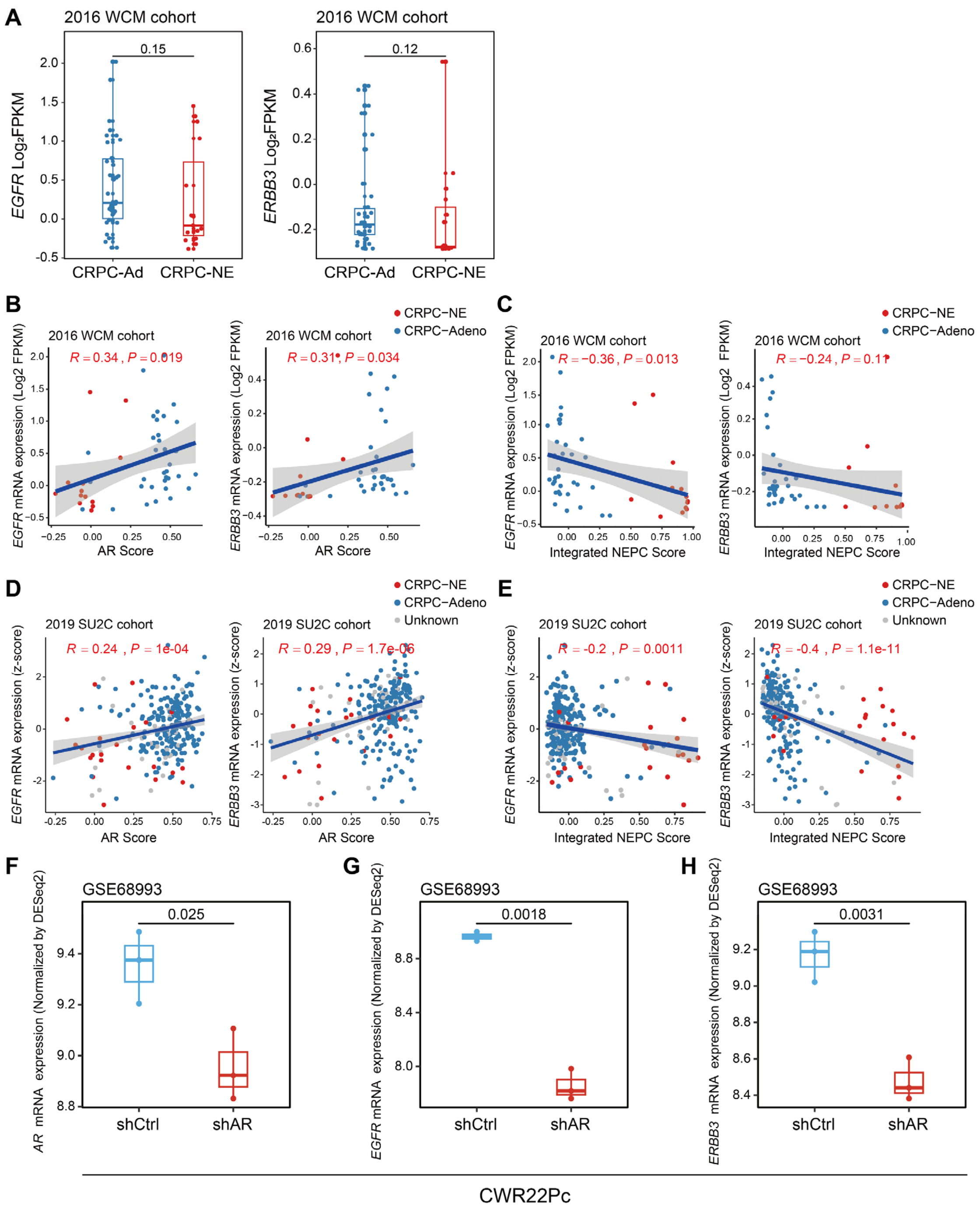

Supplemental Figure 3: Association of *EGFR* and *ERBB3* mRNA expression with neuroendocrine and androgen receptor signaling in mCRPC.

(A) Comparison of *EGFR* and *ERBB3* mRNA expression between mCRPC-Ad and mCRPC-NE tumors in the WCM cohort. The box plot represents the IQR divided by the median. Statistical significance was determined by Mann-Whitney U test.

(B-C) Pearson correlations analysis of *EGFR* and *ERBB3* mRNA levels with AR (B) and NEPC (C) signaling transcriptomic scores in the WCM cohort.

(D-E) Pearson correlations analysis of *EGFR* and *ERBB3* mRNA levels with AR (D) and NEPC (E) signaling transcriptomic scores in the SU2C cohort.

(F–H) Analysis of RNA-seq data from the GEO dataset GSE68993 showing the effect of AR knockdown on *AR* (F), *EGFR* (G), and *ERBB3* (H) mRNA expression in CWR22Pc prostate cancer cells. Data are presented as box plots indicating the interquartile range divided by the median. Statistical significance was determined by the Mann–Whitney U test.

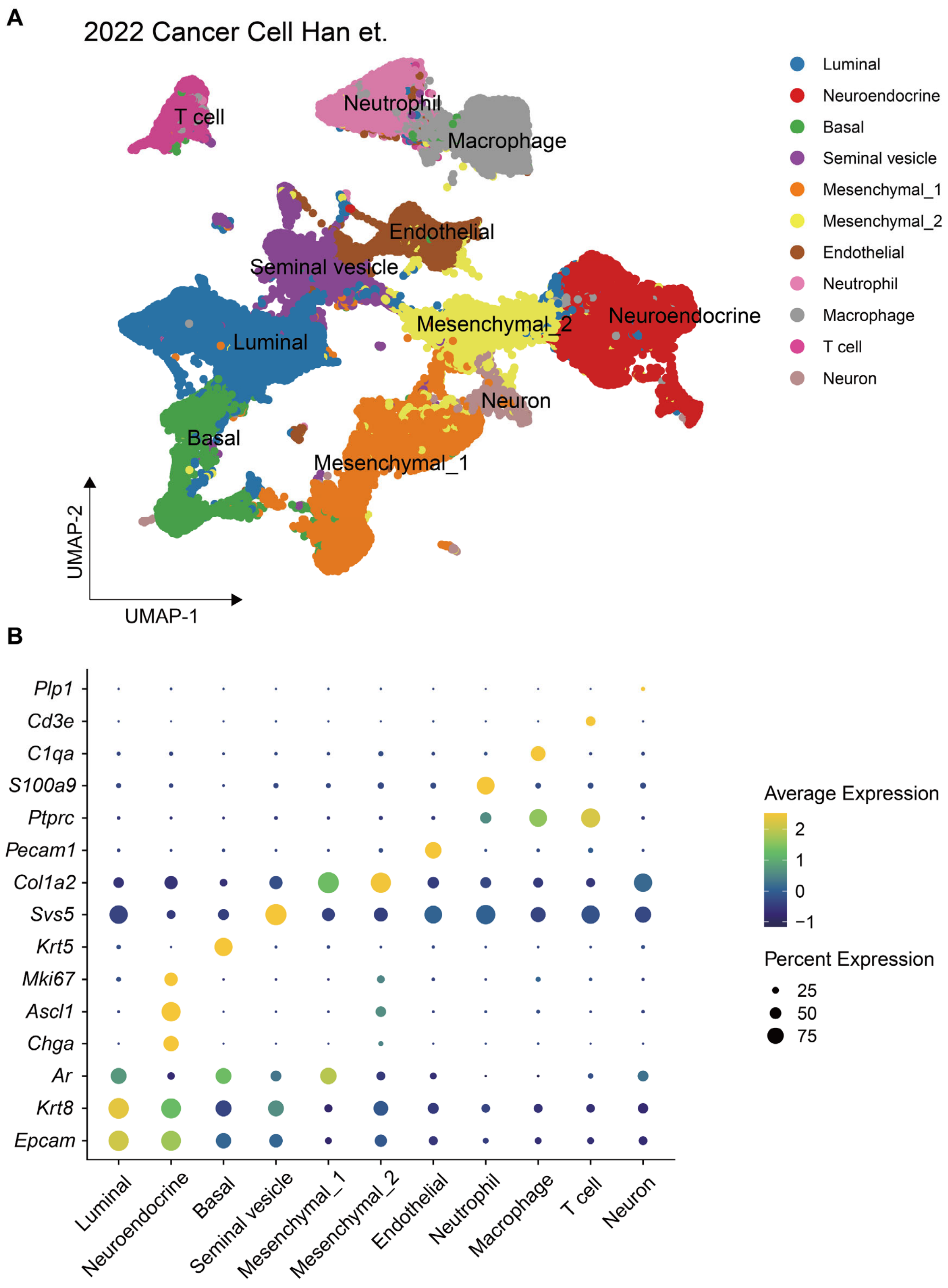

Supplemental Figure 4: Overview of cell populations and lineage annotation in a mouse model of neuroendocrine prostate cancer.

(A) UMAP visualization of cell-type clusters from a published scRNA-seq dataset of a

mouse model of neuroendocrine differentiation in prostate cancer.

(B) Dot plot showing the RNA expression levels of representative marker genes in annotated cell clusters.

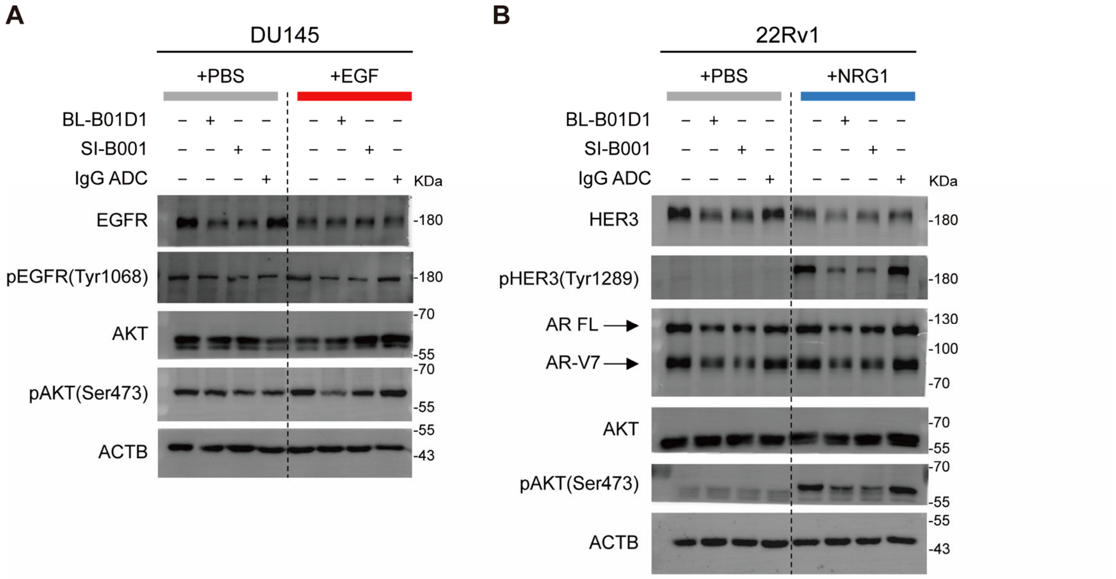

C

IC50 value (nM, ±95% CI)			
Cell line	Isotype ADC	SI-B001	BL-B01D1
LNCaP	591.2 (467.8-767.9)	1691 (1215-2608)	46.94 (41.94-52.49)
C4-2	609.8 (538.2-696.1)	4240 (2523-11199)	40.36 (33.08 -49.10)
22Rv1	583.3 (502.1-684.7)	1007 (801.2-1311)	79.38 (72.99-86.34)
PC-3	561.9 (492.4-646.4)	4098 (2062-30204)	157.1 (133.7 -185.2)
DU145	939 (764.0-1186)	2978 (2185-4509)	93.33 (84.01-103.8)

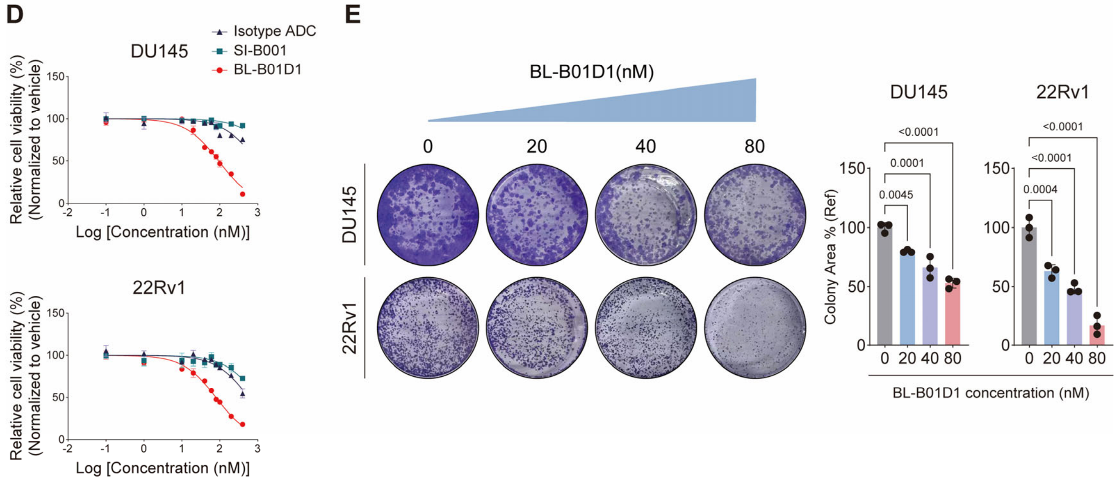

Supplemental Figure 5: In vitro evaluation of BL-B01D1 and its unconjugated antibody SI-B001 in prostate cancer cell lines.

(A-B) Western blot analysis of downstream signaling pathways in DU145 (A) and 22Rv1 (B) cells treated with PBS, BL-B01D1, the unconjugated bispecific antibody SI-B001 or isotype IgG ADC (50nmol/L, 24hours) in the presence or absence of exogenous epidermal growth factor (EGF) (100ng/mL, 10 minutes) or neuregulin-1

(NRG-1) (100ng/mL, 20 minutes).

(C) IC_{50} values (nM, ±95% CI) of BL-B01D1, SI-B001, and isotype IgG ADC across five prostate cancer cell lines (LNCaP, C4-2, 22Rv1, PC-3, and DU145).

(D) Cell viability assays showing the cytotoxic effects of BL-B01D1 compared with SI-B001 and isotype IgG ADC in DU145 and 22Rv1 cells (n = 3 biologically independent experiments). The data are shown as mean ± SD.

(E) Colony formation assays in DU145 and 22Rv1 cells treated with increasing concentrations of BL-B01D1; quantification shown at right (n = 3 biologically independent experiments). The data are shown as mean ± SD. Statistical significance was determined by one-way ANOVA with Dunnett’s multiple comparisons.

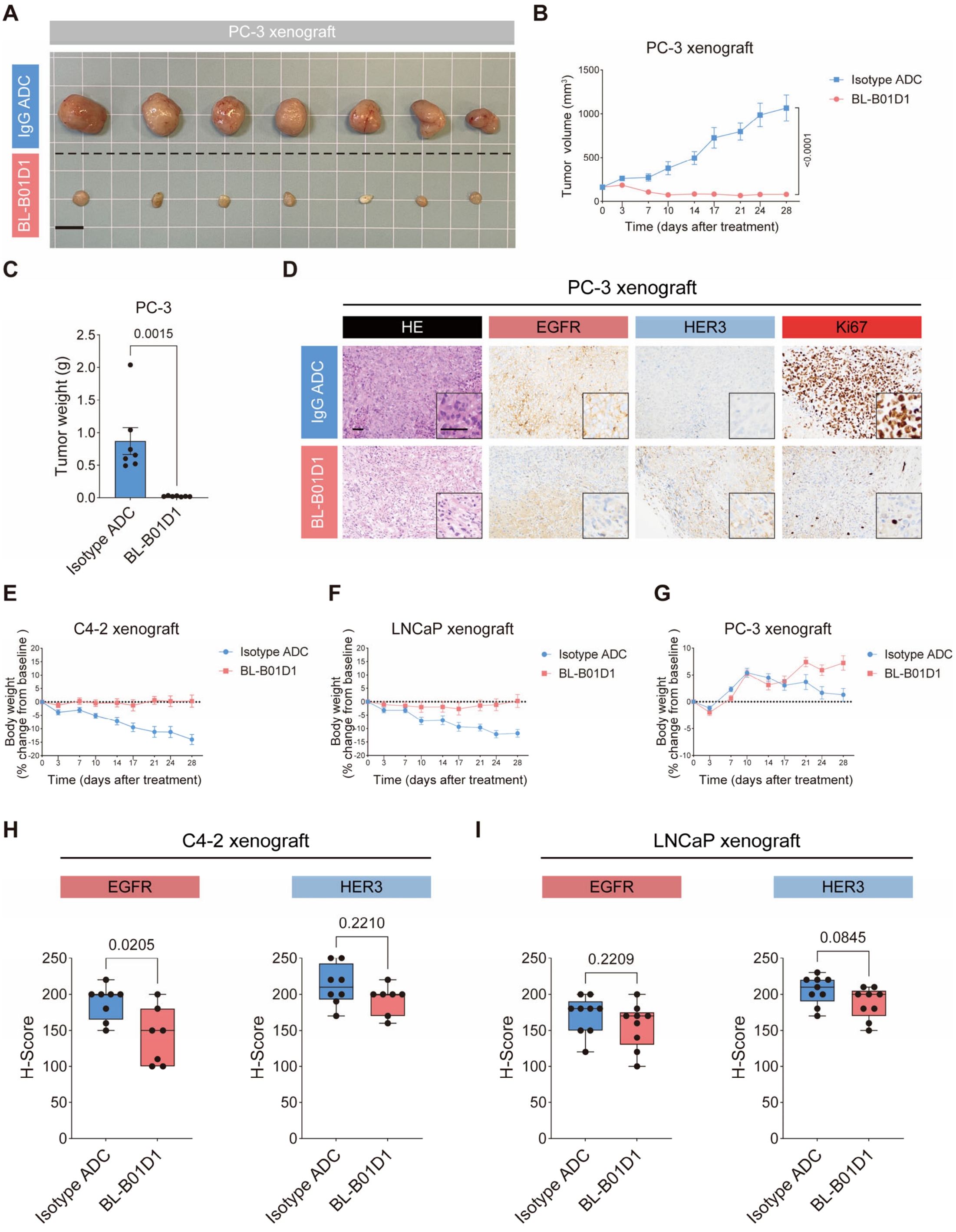

Supplemental Figure 6: Antitumor efficacy and safety evaluation of BL-B01D1 in prostate cancer xenograft models.

(A) Tumor images at the end of treatment for PC-3 xenografts. Scale bar, 1 cm.

(B) Tumor growth curves and (C) final tumor weights in PC-3 xenograft models treated with BL-B01D1 or isotype IgG ADC (n = 7 mice/group). The data are shown as mean

± SEM, Statistical significance was determined by two-tailed unpaired t test.

(D) Representative H&E and IHC staining of EGFR, HER3, and Ki67 in xenograft tumor tissues from PC-3 treated with BL-B01D1 or isotype IgG ADC (n = 7 mice/group). Scale bar, 50 μm.

(E-G) Body weight changes in mice bearing C4-2 (E), LNCaP (F), and PC-3 (G) xenograft tumors during treatment with BL-B01D1 or isotype IgG ADC (n = 8 mice/group for C4-2 xenograft, n = 9 mice/group for LNCaP xenograft, n = 7 mice/group for PC-3 xenograft).

(H-I) Semi-quantitative analysis of EGFR and HER3 expression in C4-2 (H) and LNCaP (I) xenograft tumors following Isotype ADC or BL-B01D1 treatment. The box plot represents the IQR divided by the median. Statistical significance was determined by Mann–Whitney U test.

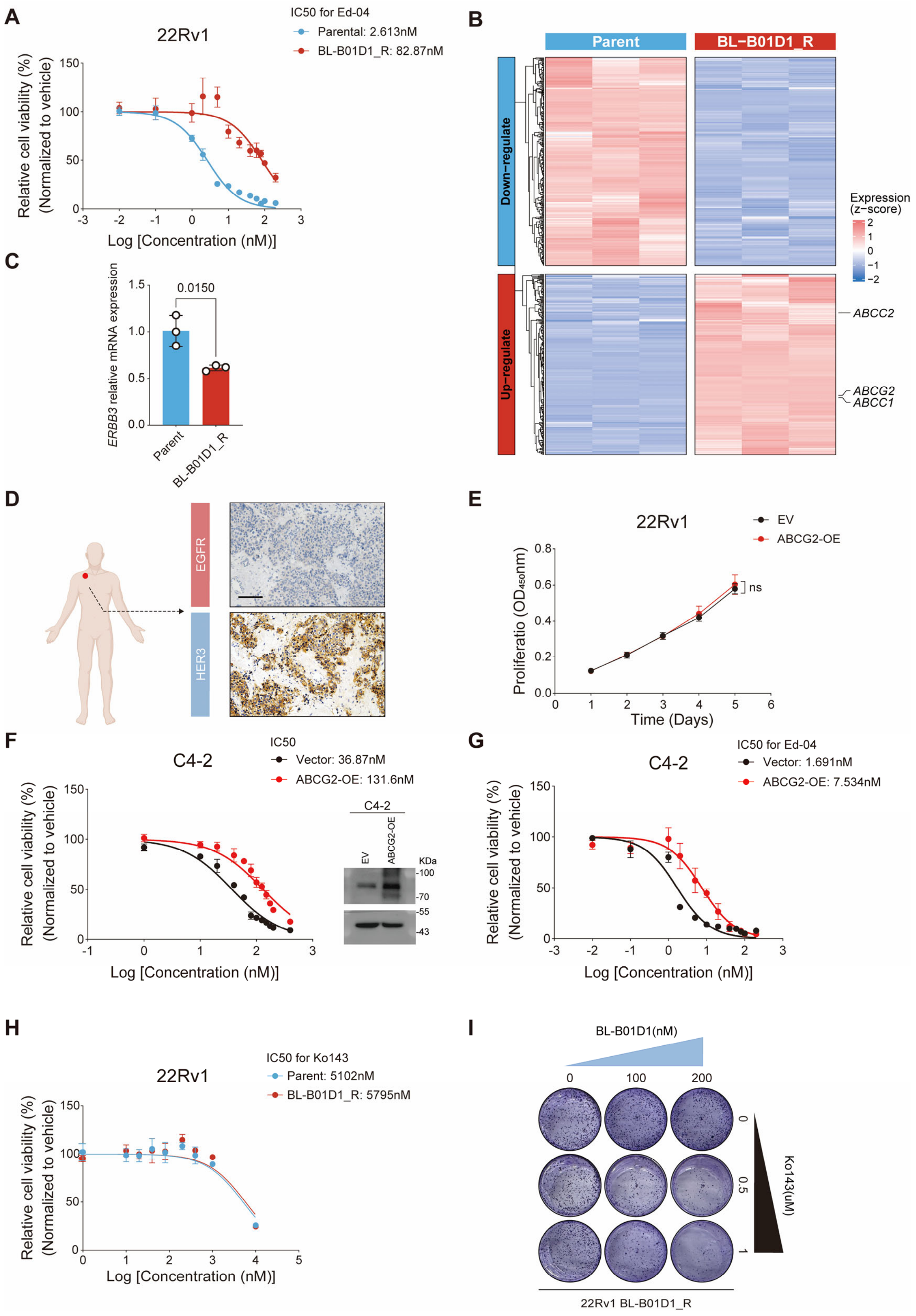

Supplemental Figure 7: ABCG2 overexpression contributes to BL-B01D1 resistance in prostate cancer cells.

(A) Cell viability assays of parental and 22Rv1 BL-B01D1_R cells treated with escalating doses of Ed-04 (n = 3 biologically independent experiments). The data are shown as mean ± SD.

(B) Heatmap of differentially expressed genes between parental 22Rv1 cells and 22Rv1 BL-B01D1_R cells, highlighting upregulation of ATP-binding cassette (ABC) transporter family members (*ABCG2*, *ABCC1*, *ABCC2*).

(C) Quantification of *ERBB3* mRNA expression in parental and resistant 22Rv1 cells by qRT-PCR. Statistical significance was determined by unpaired t test.

(D) IHC staining of EGFR and HER3 in progressive lesion from the patient who developed resistance after BL-B01D1 treatment. Scale bar, 100 μm.

(E) Cell proliferation curves of 22Rv1 cells transduced with empty vector (EV) or ABCG2 overexpression construct (ABCG2-OE). (n = 3 biologically independent experiments). The data are shown as mean ± SD. Statistical significance was determined by unpaired t test.

(F) Cell viability assays of C4-2 cells with or without ABCG2 overexpression treated with escalating doses of BL-B01D1 (n = 3 biologically independent experiments). The data are shown as mean ± SD. Corresponding Western blot results are shown.

(G) Cell viability assays of C4-2 cells with or without ABCG2 overexpression treated with escalating doses of Ed-04. (n = 3 biologically independent experiments). The data are shown as mean ± SD.

(H) Cell viability of parental and 22Rv1 BL-B01D1_R cells treated with the selective ABCG2 inhibitor Ko143. (n = 3 biologically independent experiments). The data are shown as mean ± SD.

(I) Representative images of colony formation assay of BL-B01D1_R cells treated with BL-B01D1 alone or in combination with Ko143 at the indicated concentrations (n = 3 biologically independent experiments).

SUPPLEMENTAL TABLES

Supplemental Table 1. Baseline characteristics in evaluated CRPC patients			
	**Overall (n=70)**	**CRPC-Ad (n=54)**	**CRPC-NE (n=16)**
Age at diagnosis (years)			
Median (IQR)	64.0 (58.3-69.0)	64.0 (58.3-69.8)	66.5 (60.3-69.0)
Mean (s.d.)	63.8 (7.32)	63.5 (7.57)	64.8 (6.30)
Race, n (%)			
Asian	70(100%)	54 (100%)	16 (100%)
Gleason score, n (%)			
<8	8 (11.4%)	8 (14.8%)	0 (0)
=8	14 (20.0%)	13 (24.1%)	1 (6.25%)
>8	38 (54.3%)	29 (53.7%)	9 (56.25%)
NA	10 (14.3%)	4 (7.4%)	6 (37.5%)
PSA at diagnosis (ng/mL)[#]			
Median (range)	39.0 (0.97-1540)	43.521 (0.97-1540)	23.2 (0.984-263)

IQR = interquartile range

s.d. = standard deviation

NA = not available

[#]PSA at diagnosis was not available for 11 patients (8 with CRPC-Ad and 3 with CRPC-NE). For some patients, PSA at diagnosis was reported as >100 ng/mL; a value of 100 ng/mL was used for calculation in these cases.

Supplemental Table 2. Antibodies used in this study		
**Antibodies**	**Company**	**Cat. No.**
EGFR	CST	#4267
EGFR	STARTER	#S0B2145
pEGFR	CST	#3777
ERBB3	CST	#12708
pERBB3	CST	#2842
AR	CST	#5153
SYP	Abcam	#ab32127
ABCG2	CST	#42078
AKT	CST	#4691
pAKT	CST	#4060
ACTB	Proteintech	#66009-1-Ig
HRP-conjugated Affinipure Goat Anti-Mouse IgG(H+L)	Proteintech	#SA00001-1
HRP-conjugated Affinipure Goat Anti-Rabbit IgG(H+L)	Proteintech	#SA00001-2
Universal HRP-conjugated Secondary Antibody	Abclonal	#RK50015
Ki67	CST	#9449
Anti-EpCAM Rabbit pAb	Servicebio	#GB11274-100
Anti-Cytokeratin 8 Rabbit pAb	Servicebio	#GB11231-100
Anti-Cytokeratin 5 Mouse mAb	Servicebio	#GB121246-100
FITC anti-human CD326 (EpCAM) Antibody	Biolegend	#369813
PE anti-human EGFR Antibody	Biolegend	#352904
APC anti-human erbB3/HER-3 Antibody	Biolegend	#324707
PE Mouse IgG1, κ Isotype Ctrl Antibody	Biolegend	#400113
APC Mouse IgG2a, κ Isotype Ctrl Antibody	Biolegend	#400219
Human TruStain FcX™ (Fc Receptor Blocking Solution)	Biolegend	#422301

Supplemental Table 3. Sequences of siRNAs and oligonucleotides used for shRNA and sgRNA construction	
	**Sequence (5'-3')**
siNC	UUCUCCGAACGUGUCACGUTT
siEGFR	GCCACAAAGCAGUGAAUUUAUTT
siERBB3	AAUUCUCUACUCUACCAUUGTT
shSCR	CCGGCAACAAGATGAAGAGCACCAACTCGAGTTGGTGCTCTTCACTTGTTGTTTTTG
shEGFR#1	CCGGGCCACAAAGCAGTGAATTTATCTCGAGATAAATTCACTGCTTTGTGGCTTTTTG
shEGFR#2	CCGGCGCAAAGTGTGTAACGGAATACTCGAGTATTCCGTTACACACTTTGCGTTTTTG
shHER3#1	CCGGTATATGAATCGGCAACGAGATCTCGAGATCTCGTTGCCGATTCATATATTTTTG
shHER3#2	CCGGCTTCGTCATGTTGAACTATAACTCGAGTTATAGTTCAACATGACGAAGTTTTTG
sgNT	CACCGGCCTGCCCTAAACCCCGGAA
sgABCG2#1	CACCGTATTAGATGTCTTAGCTGCA
sgABCG2#2	CACCGGCTGCAAGGAAAGATCCAAG

**Supplemental Table 4. Primers used in this study**	
**Primers used for constructing overexpression vectors (including plasmid homologous arms)**	**Sequences (5'-3')**
ERBB3-F	ATTTCAGGTGTCGTGAGGATCCAT GAGGGCGAACGACGCT
ERBB3-R	GCGGCCGCCCTCGAGGAATTCTT ACGTTCTCTGGGCATTAGCC
ABCG2-F	AGATTCTAGAGCTAGCGAATTCAT GTCTTCCAGTAATGTCGAA
ABCG2-R	GATCCTTGCGGCCGCGGATCCTTAAGA ATATTTTTTAAGAAATAACAATTTC
**Primers used for quantitative RT-PCR**	**Sequences (5'-3')**
ERBB3-F	GACCCAGGTCTACGATGGGAA
ERBB3-R	GTGAGCTGAGTCAAGCGGAG
ACTB-F	CATGTACGTTGCTATCCAGGC
ACTB-R	CTCCTTAATGTCACGCACGAT

**Supplemental Table 5. Basic composition of organoid culture medium**		
**Component**	**Company**	**Cat. No.**
Advanced DMEM/F12	Gibco	#12634-010
Penicillin/Streptomycin (100×)	Life Technologies	#15140-122
HEPES (100×)	Gibco	#15630080
Glutamax (100×)	Gibco	#35050061
B27 (50×)	Invitrogen	#17504-044
Noggin	Sino Biological Inc.	#50688-M02H
R-spondin 1	Sino Biological Inc.	#11083-HNAS
N-acetylcysteine	Solarbio	#C8460-5g
EGF	Sino Biological Inc.	#50482-MNCH
Human bFGF	PeproTech	#GMP100-18B-25UG
Human FGF10	PeproTech	#100-26-25UG
(DiHydro) testosterone (5α-Androstan-17β-ol-3-one)	Merk	#A8380
Nicotinamide	Solarbio	#N8070-25g
A83-01	GLPBIO	#GC10166
Prostaglandin E2	Solarbio	#IP3180

SUPPLEMENTAL REFERENCES

1. Ianevski A, Giri AK, and Aittokallio T. SynergyFinder 3.0: an interactive analysis and consensus interpretation of multi-drug synergies across multiple samples. *Nucleic Acids Res.* 2022;50(W1):W739–w43.
