# Supplementary material for: EGFR- and HER3-targeted bispecific antibody-drug conjugate demonstrates antitumor activity in metastatic castration-resistant prostate cancer
Source: J Clin Invest. 2026 Apr 7;136(11):e201090. doi: 10.1172/JCI201090 (PMC13221231; doi:10.1172/JCI201090)

Figure 3A

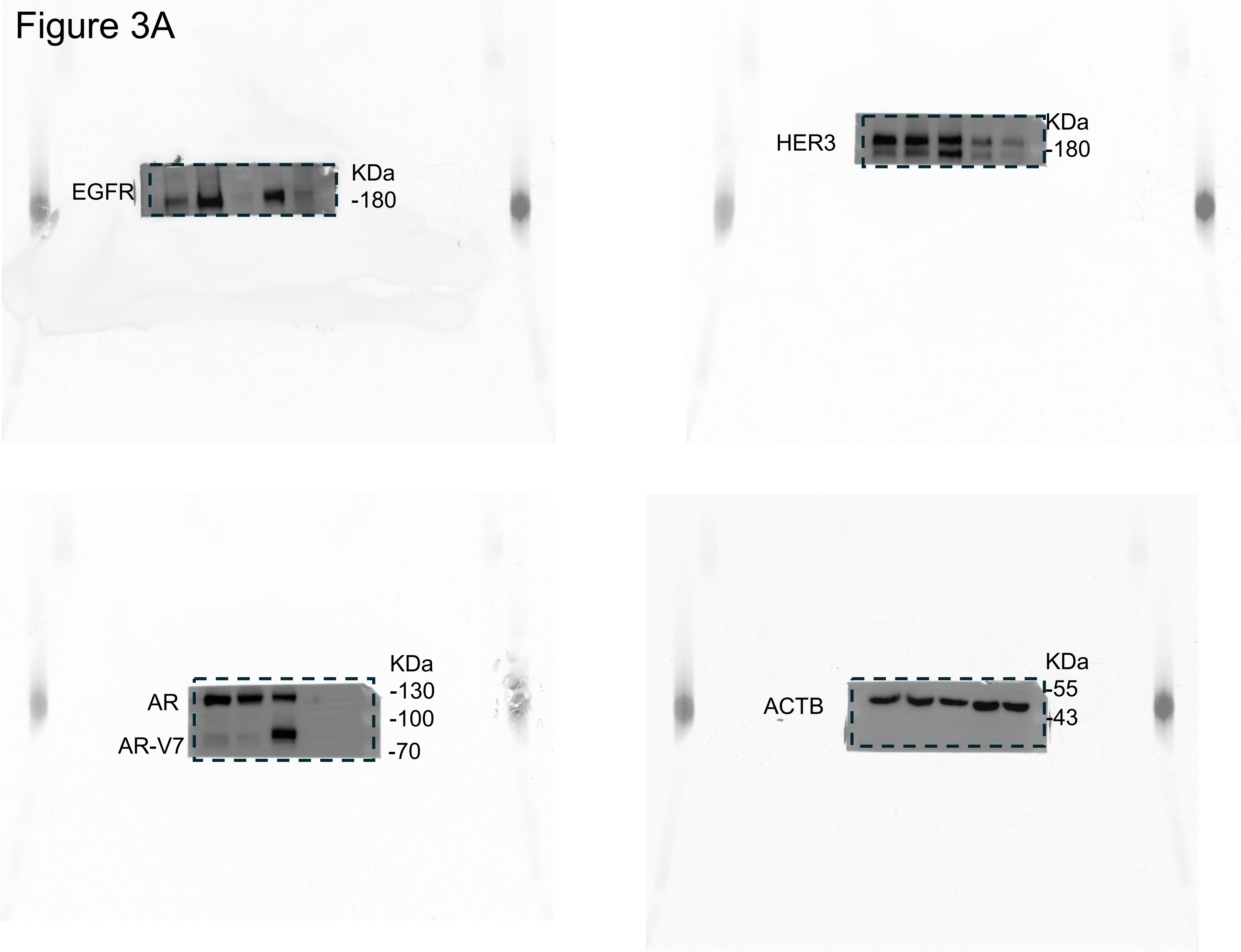

Figure 3C

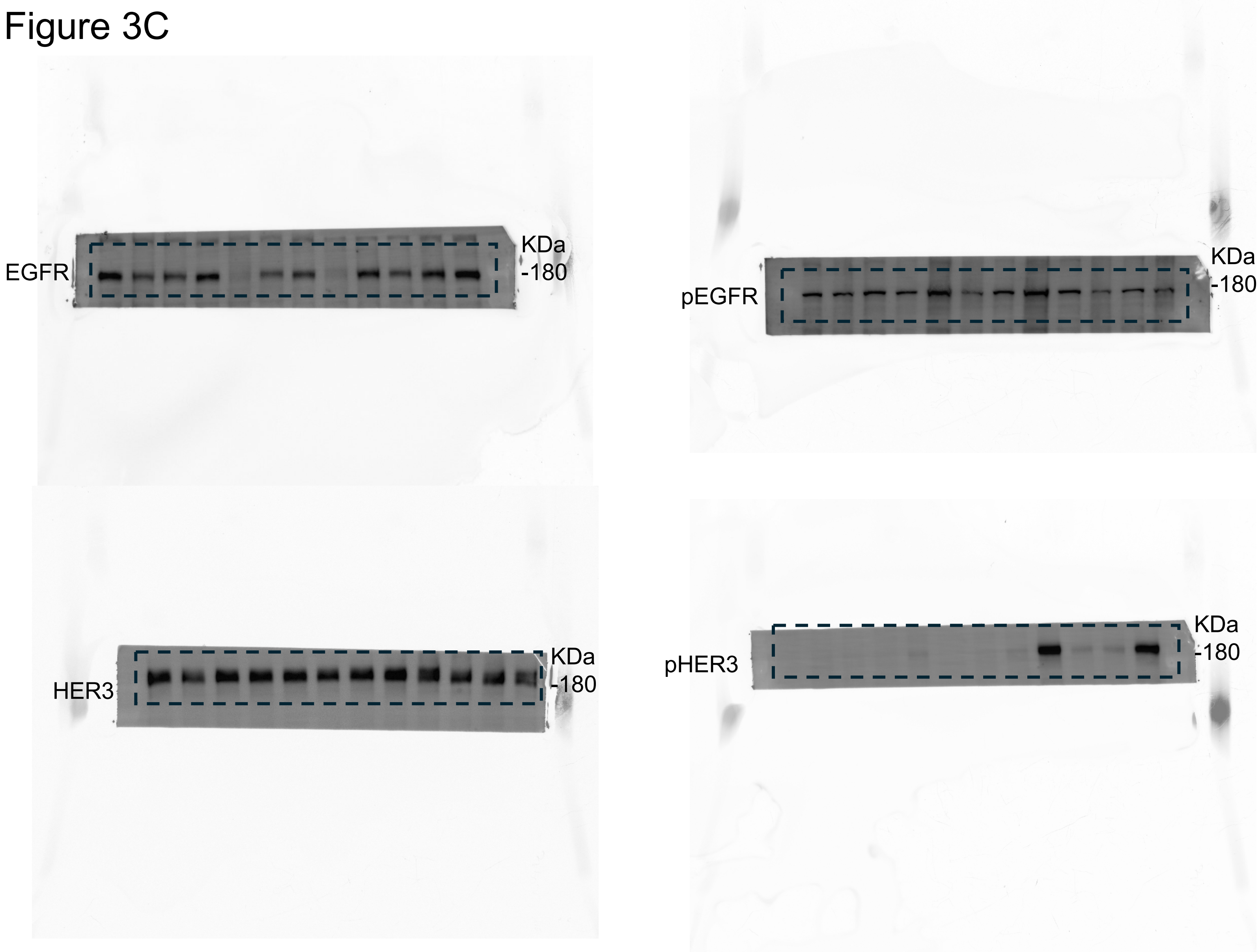

Figure 3C

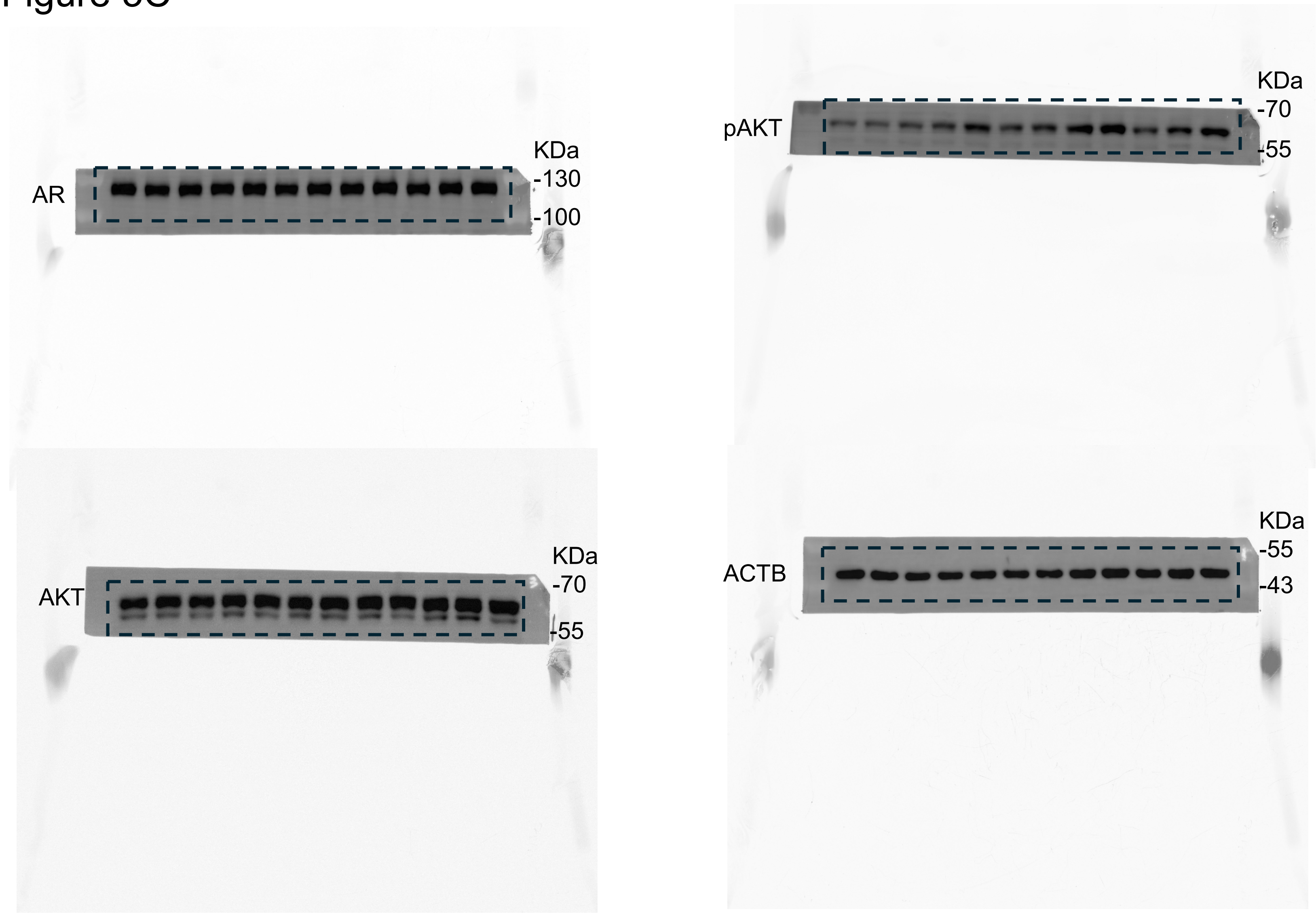

Figure 3F

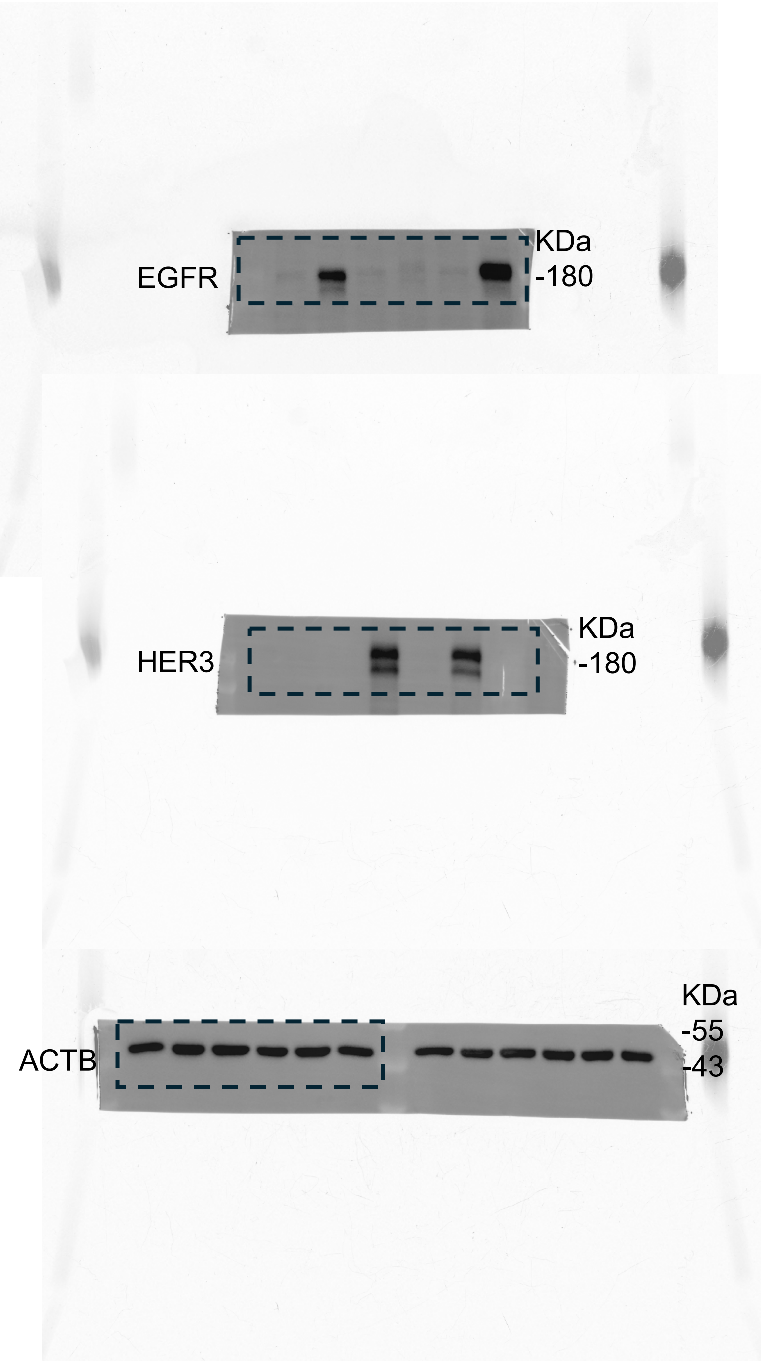

Figure 3G

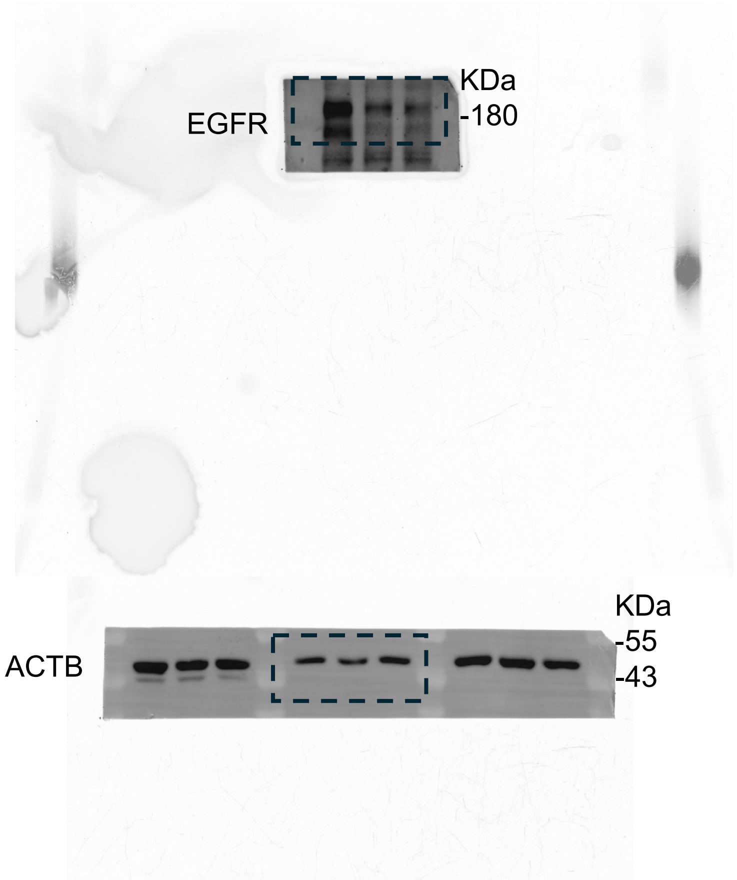

Figure 3H

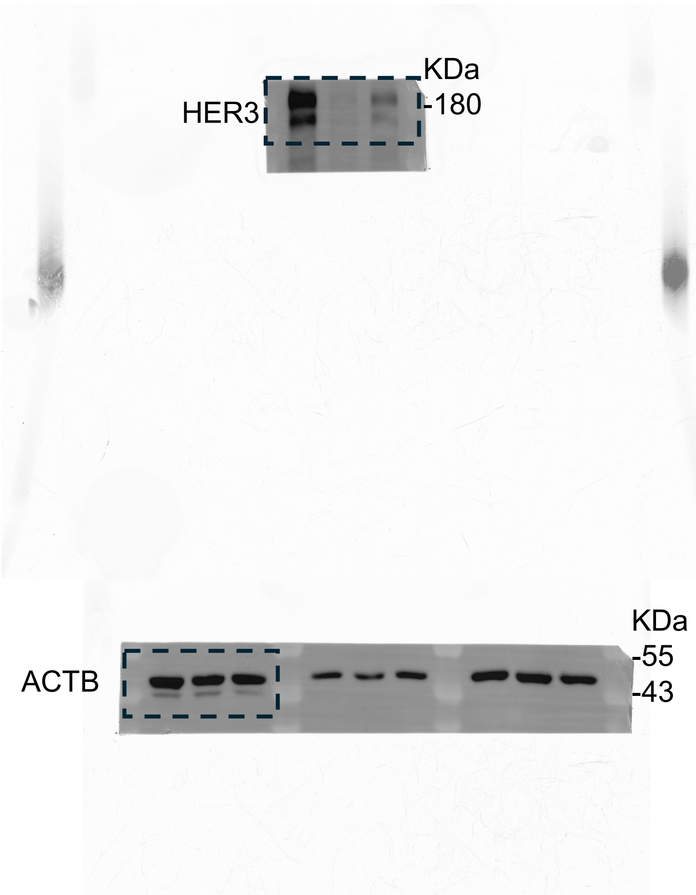

Figure 7F

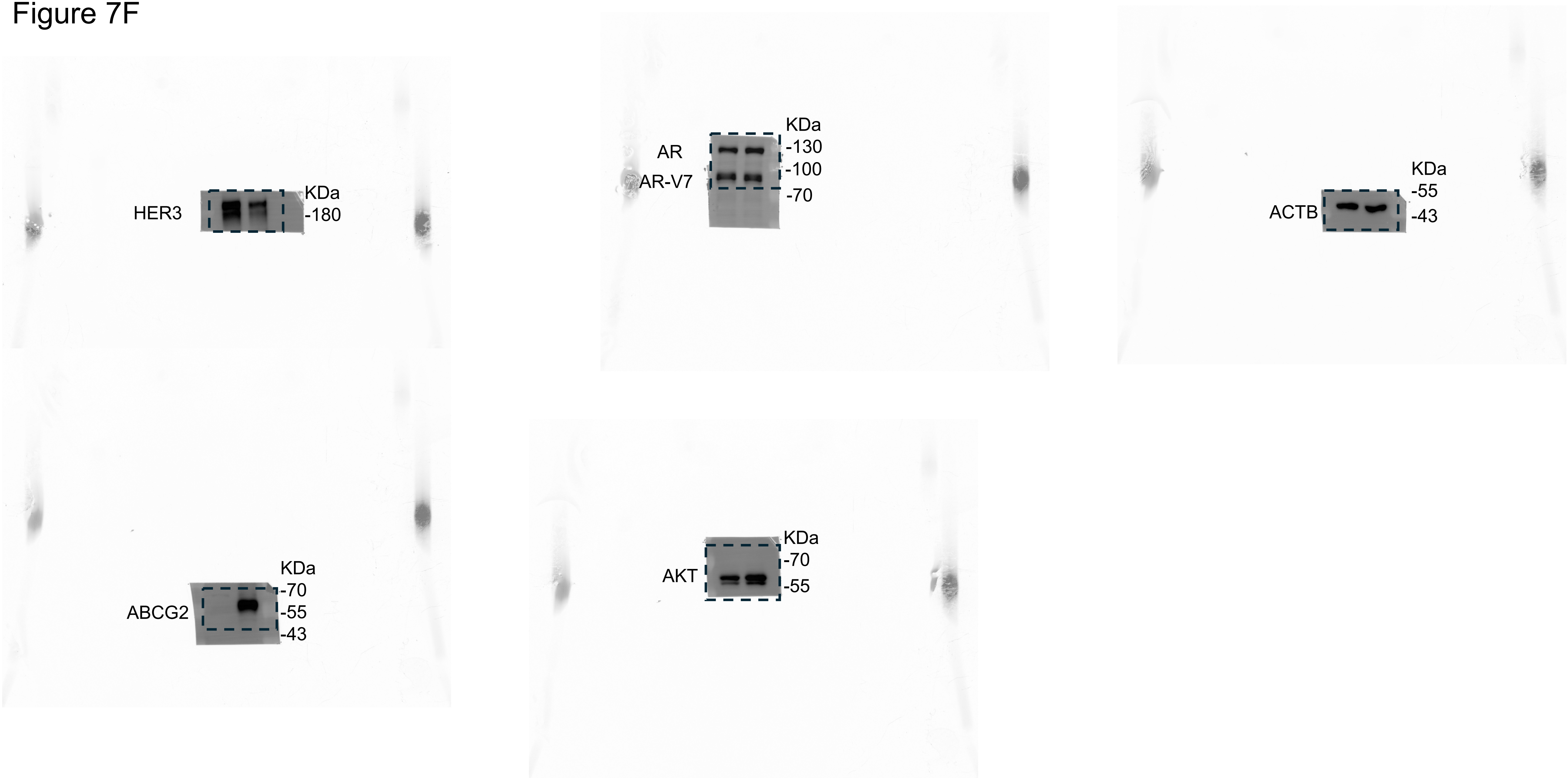

HER3
KDa
-180
AR
AR-V7
KDa
-130
-100
-70
ACTB
KDa
-55
-43
ABCG2
KDa
-70
-55
-43
AKT
KDa
-70
-55

Figure 7H

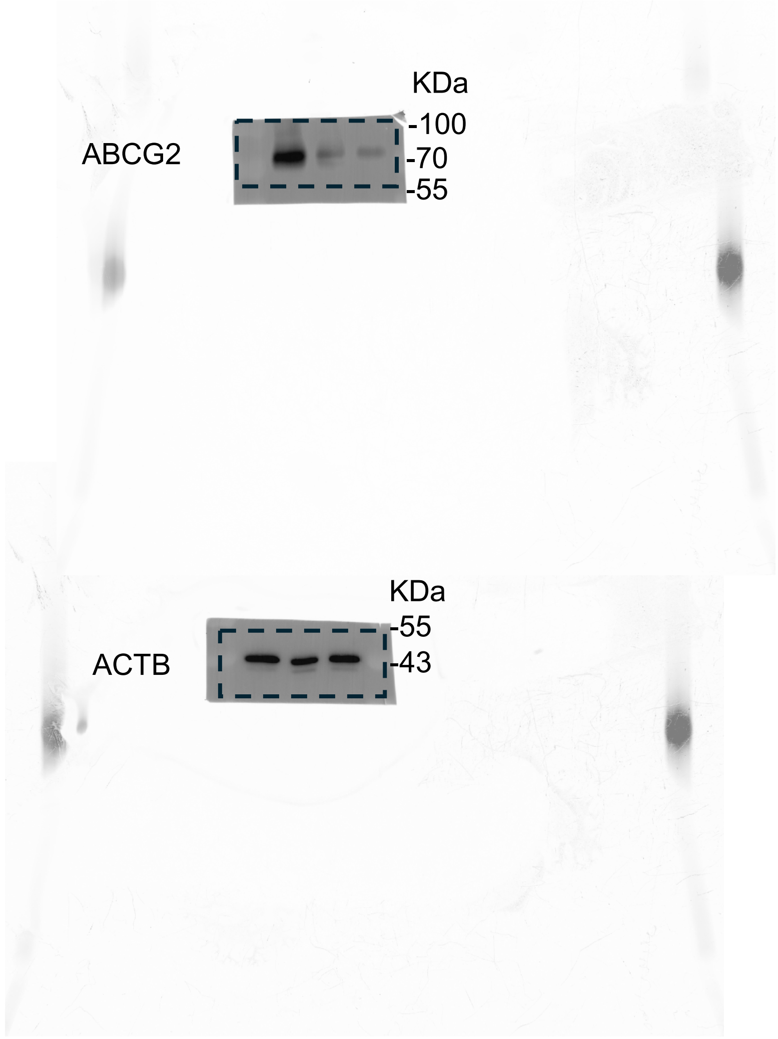

Figure 7I

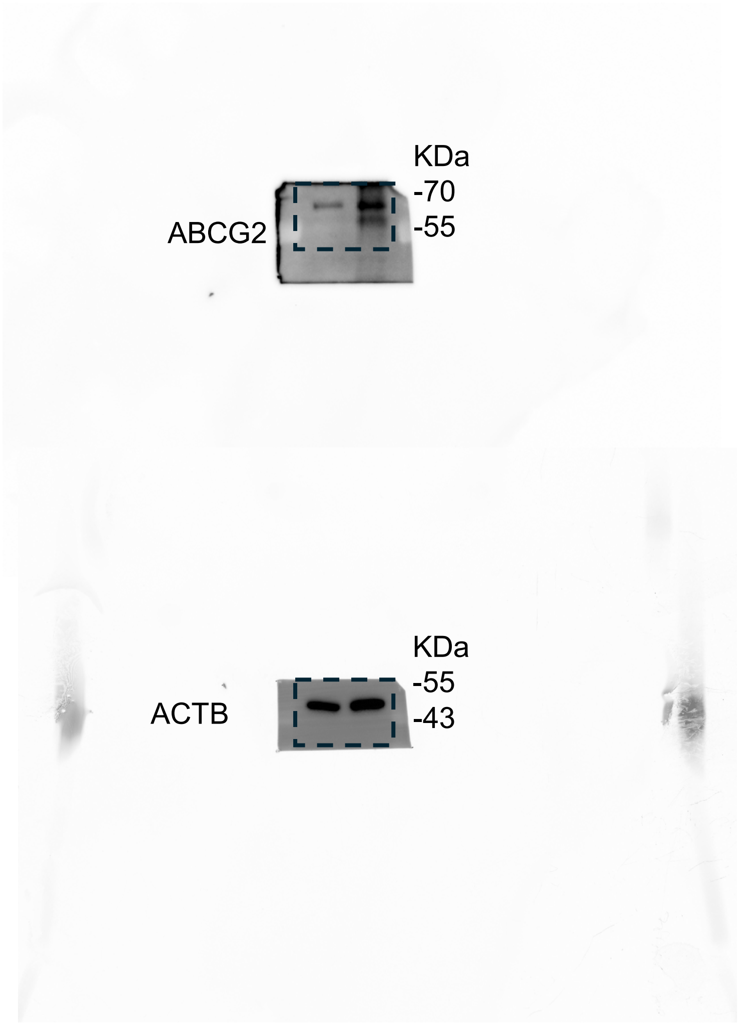

Supplemental Figure 2A

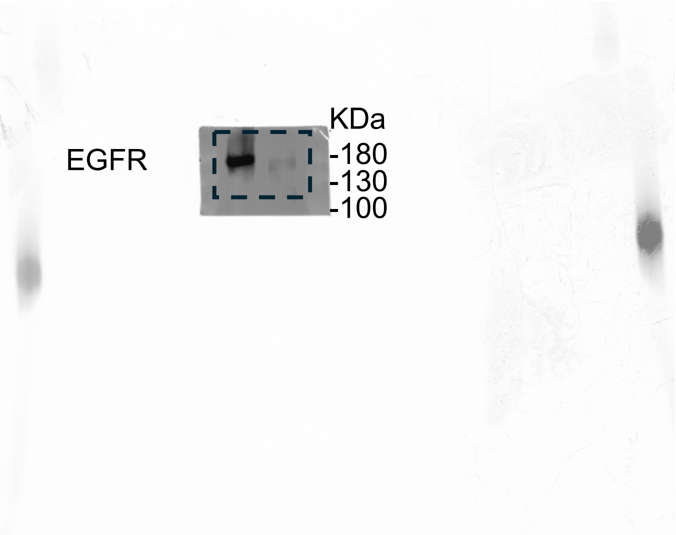

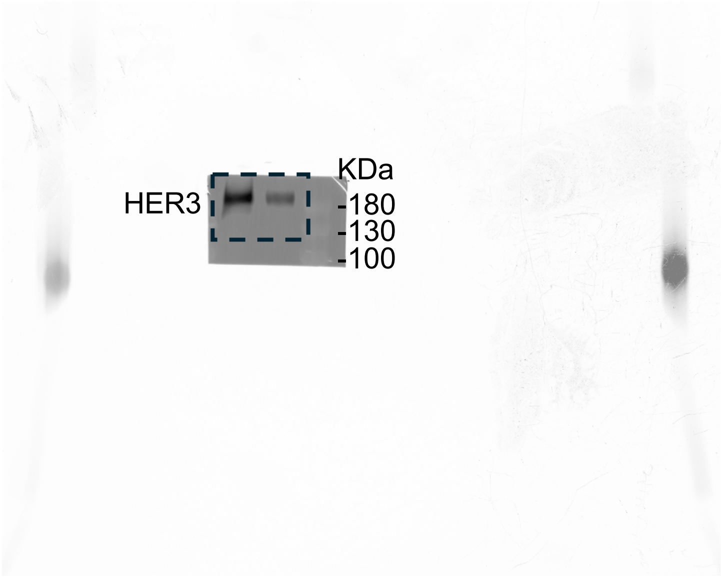

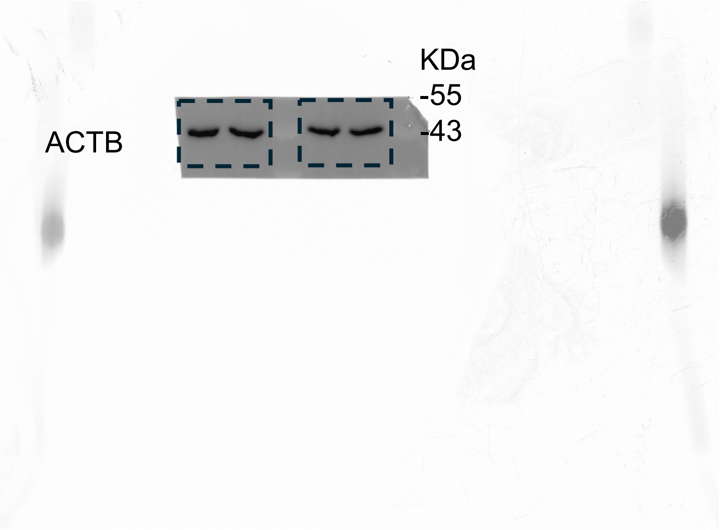

Supplemental Figure 5A

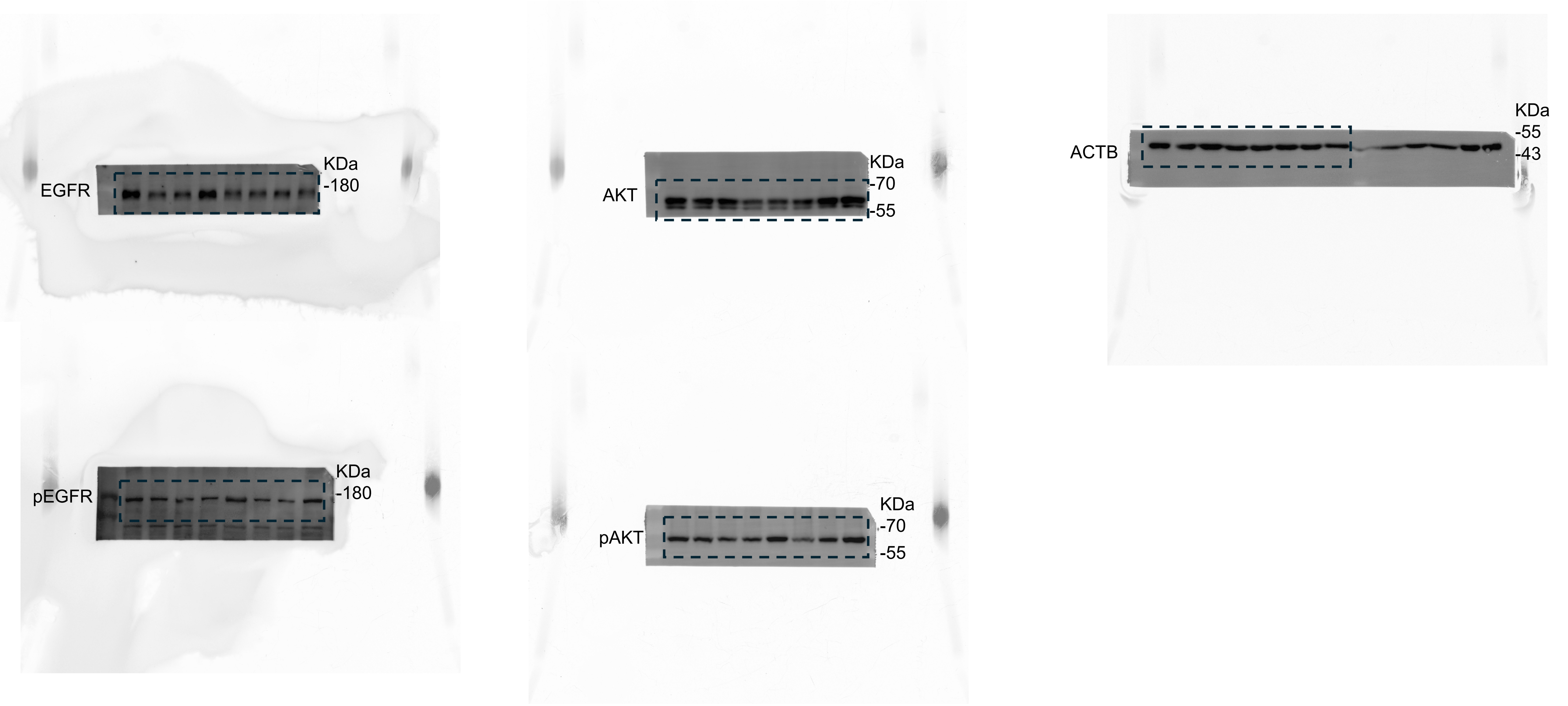

Supplemental Figure 5B

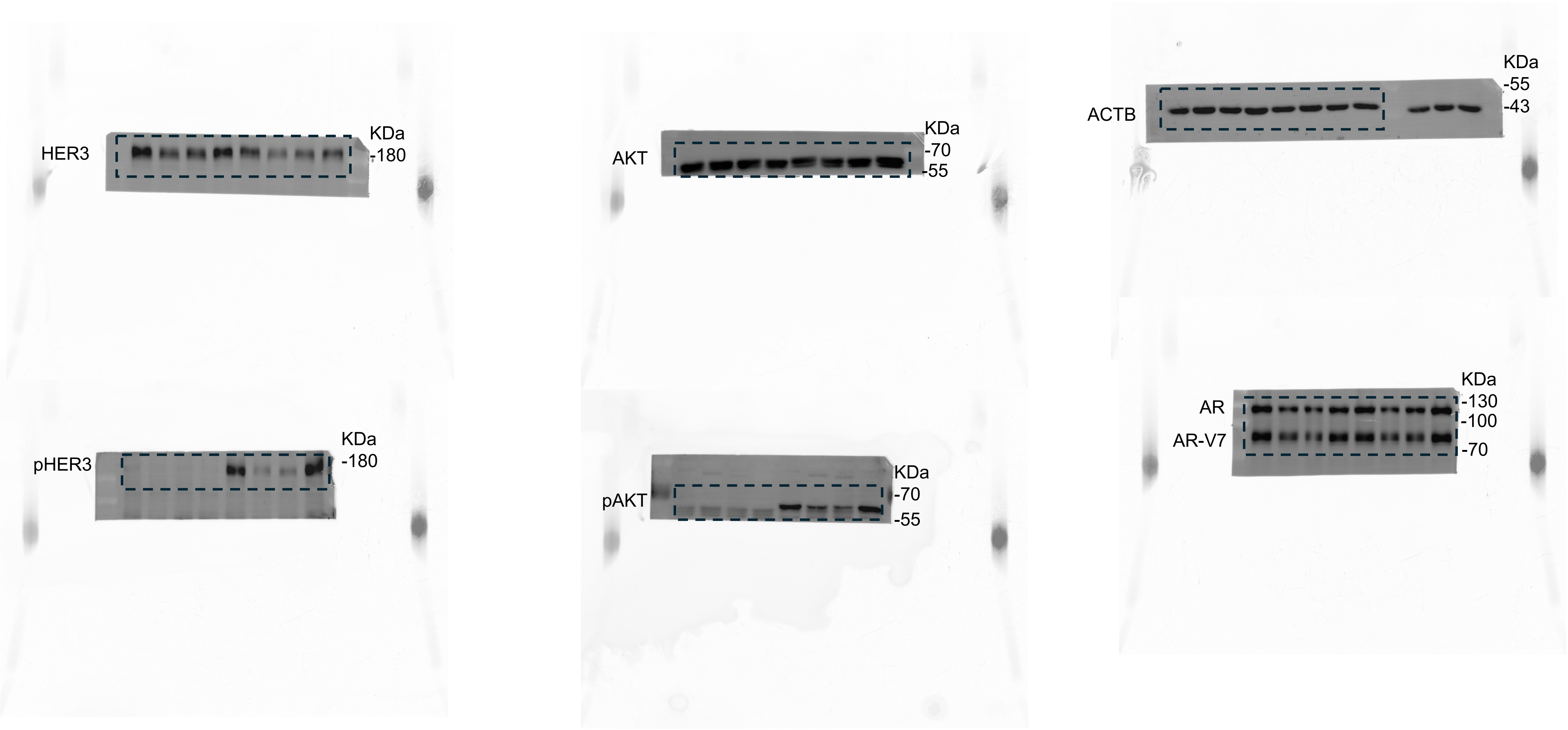

Supplemental Figure 7F

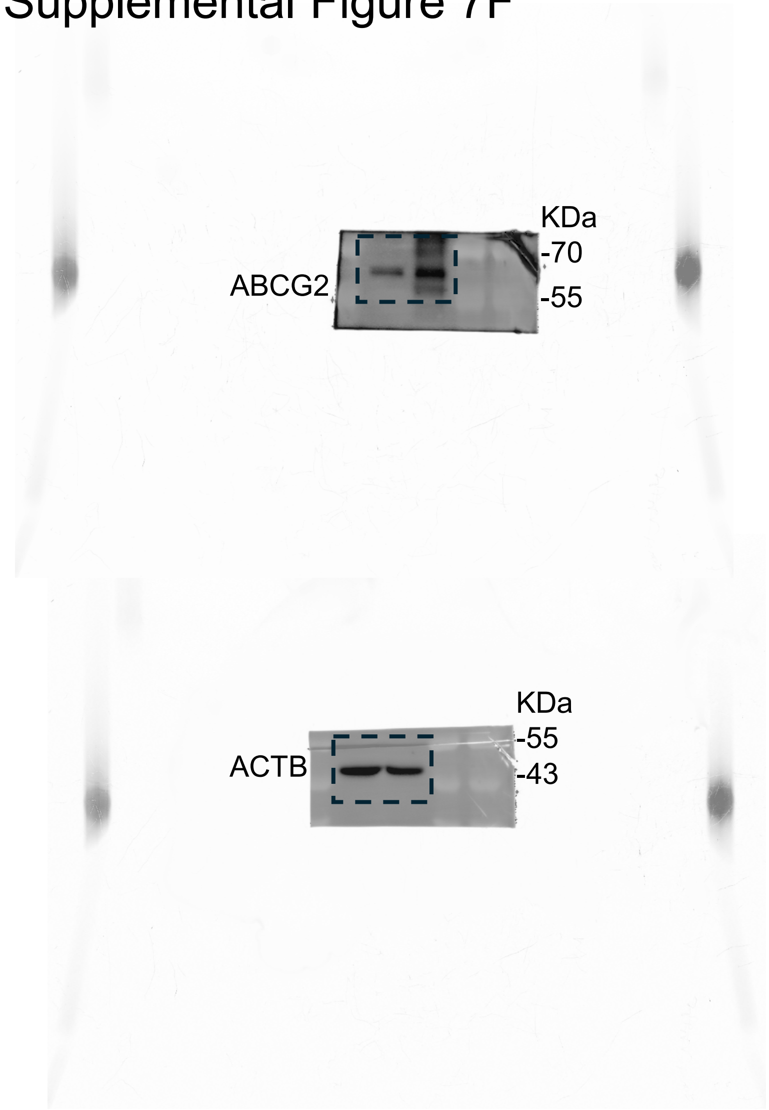

Supplement: Unedited blot and gel images [file jci-136-201090-s052.pdf]
